# Serendipity and the Slime Mold: A Visual Survey of High-Molecular-Weight Protein Assemblies Reveals the Structure of the Polyketide Synthase Pks16

**DOI:** 10.1016/j.mcpro.2025.101484

**Published:** 2025-12-09

**Authors:** Gabriel Hoogerbrugge, Adrian T. Keatinge-Clay, Edward M. Marcotte

**Affiliations:** Department of Molecular Biosciences, The University of Texas at Austin, Austin, Texas, USA

**Keywords:** Structural Proteomics, cryo-electron microscopy, polyketide synthase, slime mold, shotgun electron microscopy

## Abstract

Large macromolecular assemblies are integral to most cellular processes, making their identification and structural characterization an important strategy for advancing our understanding of protein functions. In this pilot study, we investigated large multiprotein assemblies from the cytoplasm of the slime mold *Dictyostelium discoideum* using shotgun electron microscopy, the combined application of mass spectrometry–based proteomics and cryo-EM to heterogenous mixtures of proteins. With its similarities in cell structure and behavior to mammalian cells, *D. discoideum* has long served as an invaluable model organism, particularly in the study of immune cell chemotaxis, phagocytosis, bacterial infection, and other processes. We subjected *D. discoideum* soluble protein complexes to two-step fractionation, performing size-exclusion chromatography followed by mixed-bed ion-exchange chromatography. Isolated fractions containing a subset of high molecular weight-scale protein assemblies were subsequently analyzed using mass spectrometry to identify the proteins and cryo-EM to characterize their structures. Mass spectrometry analysis revealed 179 unique proteins in the isolated fractions, then single-particle cryo-EM analysis generated distinct 2D projections of several visually distinctive protein assemblies, from which we successfully identified and reconstructed three major protein complexes: the 20S proteasome, the dihydrolipoyllysine-residue succinyltransferase (Odo2) of the mitochondrial 2-oxoglutarate dehydrogenase complex, and polyketide synthase 16 (Pks16), thought to be the primary fatty acid synthase of *D. discoideum*. Based on the Pks16 structure, the first of the 40 *D. discoideum* PKSs to be experimentally determined, models for the full set of *D. discoideum* PKSs were constructed with help from AlphaFold 3. Comparative analysis enabled structural characterization of their reaction chambers. Shotgun EM thus provides a view of proteins in their native or near-native biological conformations and scaling up this approach offers an effective route to characterize new structures of multiprotein assemblies directly from complex samples.

## Highlights

• Surveyed native extracts of the slime mold *Dictyostelium* by cryo-electron microscopy.
• Observed multiple high molecular weight multiprotein assemblies.
• Solved experimental structure of *Dictyostelium* polyketide synthase (Pks16) at 3.9 Å.
• Performed a comparative structural analysis of all 40 *Dictyostelium* PKSs.

## Graphical Abstract

**Figure:**
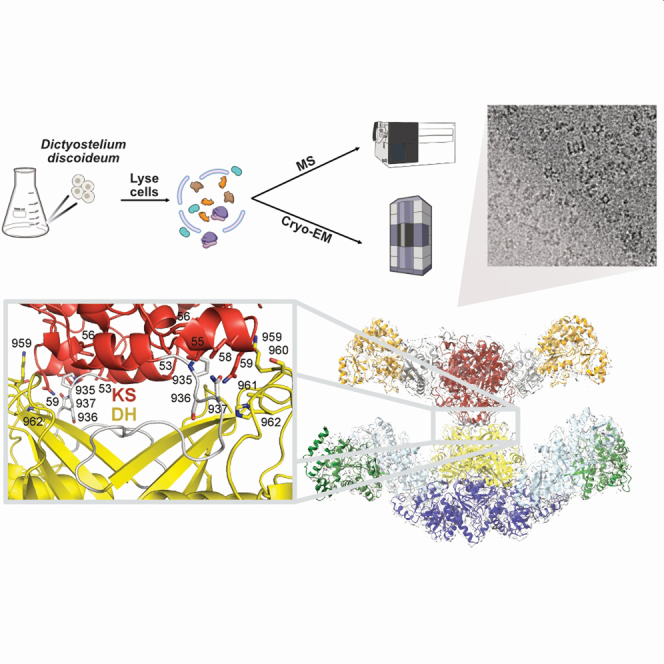

## In Brief

Large protein complexes drive essential cellular processes, yet many remain structurally uncharacterized. By combining native chromatography, cryo-electron microscopy, and mass spectrometry, we analyzed a sampling of endogenous high molecular weight protein assemblies from the slime mold *Dictyostelium discoideum*. This study uncovered multiple protein structures, including that of Pks16, its primary fatty acid synthase, enabling a comparative analysis across its full repertoire of 40 polyketide synthases.

*Dictyostelium discoideum*, a soil-dwelling social amoeba commonly referred to as slime mold, has played a pivotal role in advancing our understanding of essential biological processes, including bacterial infection, immune cell chemotaxis, phagocytosis, and mitochondrial and neurological disorders. *D. discoideum* is an excellent organism for investigating diverse aspects of eukaryotic cell biology, as it exhibits structural and behavioral characteristics akin to mammalian cells and shares conserved intracellular signaling pathways (1), while also having rich repertories of genes relevant to its alternate unicellular/multicellular lifestyle and bacterial predation activities. Due to its extensive repertoire of conserved functions, *D. discoideum* also serves as an accessible system for investigating human protein orthologs, situated within a complexity range intermediate between *Saccharomyces cerevisiae* and higher multicellular eukaryotes (1).

Of particular note, *D. discoideum* contains a rich repertoire of genes encoding polyketide synthases (PKSs), highlighting a potentially active secondary metabolic network involved in the biosynthesis and export of small molecules (2). PKSs are responsible for the synthesis of polyketides, which can have antibiotic and signaling activities. These compounds have significant applications in the pharmaceutical, agrochemical, and biotechnological industries (3, 4). Although a wide range of PKS genes are present in eukaryotic organisms, the ecological functions of the resulting polyketides—beyond their pharmaceutical utility—remain widely underexplored. Understanding the ecological roles of polyketides is essential for advancing our comprehension of microbial cellular processes and their interactions within the environment (5). *D. discoideum* has approximately 40 putative PKS genes (2), but the functional roles and structural characteristics of these PKSs are poorly understood, as only a limited number of their products have been isolated and characterized, and no structures of *Dictyostelium* PKSs have been solved to date.

There are numerous advanced techniques for isolating specific proteins of interest, including recombinant expression and affinity- or immuno-purification. However, methods that rely on protein tagging or antibodies have certain drawbacks. Specifically, the biological system must be receptive to genetic manipulation, and the addition of tags may destabilize protein–protein interactions (6) or alter protein stoichiometry, while immunopurifications require available antibodies, often lacking in less studied species. In contrast, structural analysis of endogenous protein complexes offers a promising alternative, as it bypasses the challenges posed by recombinant expression (7) and tagging techniques. Such "tagless" approaches to isolating protein assemblies can permit the unbiased analysis of cellular lysates, providing a more natural representation of protein interactions (6).

One such “tagless” approach involves directly examining endogenous protein complexes using electron microscopy (7, 8, 9, 10), which has revolutionized the structural characterization of large, flexible, and heterogeneous biomolecular complexes. The "resolution revolution," driven by the advent of direct detectors that enhance the signal-to-noise ratio in electron micrographs, along with advancements in automation for data acquisition and image processing, has enabled cryo-EM to be applied effectively for studying molecular organization in both highly purified isolated biomolecules and *in situ* (11, 12). However, it is still challenging to apply cryo-EM to heterogenous mixtures, as increased sample complexity complicates identifying individual assemblies and assigning individual particles to their corresponding structures, and the corresponding lower particle counts can lead to lower-resolution reconstructions.

Nonetheless, an emerging field within structural proteomics focuses on developing and improving methods to investigate molecular structures directly within native cell extracts. In particular, mass spectrometry (MS) provides a powerful complementary approach to determine the molecular composition of the samples. Its high sensitivity, specificity, and ability to generate both structural and quantitative data make MS particularly valuable when integrated with cryo-EM. This combined approach, sometimes referred to as shotgun EM (9), allows for the acquisition of high-resolution structures of multiple, unrelated protein complexes from a single cryo-EM dataset (7, 13). Recent studies have demonstrated the potential of this approach for resolving heterogeneous membrane protein complexes directly from cell lysates, employing lipid nanodiscs for stabilization (14, 15). This methodology has been extended to isolate and resolve multisubunit complexes from subcellular compartments, including the respiratory chain complexes of the inner mitochondrial membrane (16). Other examples include the high-resolution structural determination of nucleosomes from *Xenopus* egg extracts (17), multiple protein complexes essential for intraerythrocytic survival of *Plasmodium falciparum* (18), and structural elucidation of the yeast L-A virus capsid, highlighting cation–π interactions and capsomere interfaces from native viral preparations (19). Notable advances in this field have incorporated enzymatic activity measurements (20, 21, 22), integrated AI-based tools such as AlphaFold and FindMySequence for interpretive modeling of cryo-EM maps (23), and resolved endogenous substrate-bound states of an isomerase to 2.4 Å resolution (24)—all using cryo-EM directly from native cellular extracts. When applied to crude extracts of intermediate complexity, such approaches can enhance *in situ* techniques, such as cryo-focused ion beam cryo-electron tomography, by directly correlating structural data with molecular signatures seen in tomograms.

This study represents a pilot investigation of employing the shotgun EM approach on *D. discoideum* cell lysates in order to characterize several of its high molecular weight multiprotein assemblies. We integrated MS for protein identification with cryo-EM as a structural screening tool, enabling the generation of coarse molecular envelopes. Protein complexes were fractionated by macromolecular size via size-exclusion chromatography (SEC), followed by separation based on surface charge using mixed-bed ion-exchange chromatography (IEX). Using this integrated approach, we reconstructed four distinct protein complexes, including one yet to be identified. Notably, we serendipitously solved a 3.94 Å structure of the first *D. discoideum* PKS, Pks16. Pks16 is thought to be the primary fatty acid synthase (FAS) of *D. discoideum* (25). Hence, the structure provides an initial experimental view of this important protein family in *D. discoideum*, enabling a comparative structural analysis across the family members.

## Experimental Procedures

### Experimental Design and Statistical Rationale

A series of biological replicates were used to establish reproducible chromatographic separation and enrichment procedures. For the sample ultimately analyzed by cryo-EM (a pool of three HPLC fractions, Supplemental Fig. S1), protein MS analysis of the identical sample was performed as a technical triplicate experiment and confirmed with analysis of the three corresponding fractions from a biological replicate. MS proteomics data were analyzed with Proteome Discoverer (Thermo Fisher Scientific) as detailed below, controlling protein and peptide identification false discovery rates at <1% FDR, and are reported in Supplemental Table S1. Cryo-EM statistics were computed with cryoSPARC v4.5 (26) and are detailed in full in Supplemental Table S2 and Supplemental Figs. S2–S5, including micrograph and particle counts, 3D reconstruction processing statistics (including Fourier shell correlation (FSC) thresholds and computed map resolutions), and model refinement and validation statistics as calculated by Phenix (27). 3D structural models were computed initially with AlphaFold 3 (28) before refining subsequently with Namdinator (29).

### *Dictyostelium* Strain and Culture

*D. discoideum* AX2-214 cells, obtained from the Dicty Stock Center (DSC) at Columbia University, New York, were cultured in HL5 axenic medium (Formedium), supplemented with 74.8 mM glucose, 10,000 units/ml of penicillin, and 10,000 μg/ml of streptomycin. Cultures were maintained at 22 °C in cell culture plates, with shaking at 180 rpm in culture flasks.

### *Dictyostelium* Lysis and Soluble Protein Extraction

*D. discoideum* cultures were grown in 3 L of medium to a cell density not exceeding 4 × 10^6^ cells/ml. Cells were harvested by centrifugation at 500*g* for 4 min at room temperature. The resulting cell pellets were resuspended in 400 ml of 50 mM Hepes, pH 7.4, repelleted, and then suspended in 8 ml of Dicty Lysis Buffer (50 mM Hepes, pH 7.4, 100 mM NaCl, 3 mM MgSO_4_, 0.1 mM EGTA, 1 mM DTT, 0.1 mM PMSF, cOmplete mini EDTA-free protease inhibitors (Roche), PhosSTOP EASY phosphatase inhibitors (Roche), and 1% Igepal CA-630 (Sigma-Aldrich)). Cells were lysed by incubation on ice with vortexing every minute. We previously evaluated mechanical lysis by sonication and found that detergent-based lysis achieved comparable results while offering a simpler workflow for our application. To degrade nucleic acids, benzonuclease was added to a final concentration of 1 unit/ml and incubated on ice for 30 min. Following incubation, the lysate was centrifuged at 17,000*g* for 20 min at 4 °C to remove intact cells, intact organelles, and insoluble proteins. The supernatant was then subjected to ultracentrifugation at 100,000*g* for 1.25 h at 4 °C to remove membrane fragments and residual debris. The protein concentration in the detergent-solubilized fraction was quantified using a DC Bradford Assay (Bio-Rad) with a protein concentration yield of 14 to 18 mg/ml.

### *Dictyostelium* Soluble Protein Separation

Two milliliters of clarified soluble extract in Hepes-Dicty Lysis Buffer were fractionated by SEC with a preparative-grade HiLoad 16/600 Superdex 200 PG column (Cytiva) at a flow rate of 1 ml/min. The chromatogram shown in Supplemental Fig. S1*A* presents the complete SEC elution profile, displayed as a triplicate and monitored by absorbance at 280 nm. The mobile phase consisted of 50 mM Hepes, pH 7.4, 100 mM NaCl, and 3 mM MgSO_4_, 0.1 mM EGTA. Fractions of 1.5 ml were collected from the high molecular weight region, pooled, and concentrated using a Sartorius Vivaspin Turbo 100,000 MWCO ultrafiltration unit. The concentrated pooled fractions from the high molecular weight region were further fractionated using a mixed-bed ion exchange column (PolyLC Inc, #204CTWX0510) with a Dionex UltiMate 3000 HPLC system. Chromatographic separation was achieved by applying a gradient of buffer A (50 mM Hepes, pH 7.4, 3 mM MgSO_4_, 0.1 mM EGTA) and buffer B (50 mM Hepes, pH 7.4, 1.5 M NaCl, 3 mM MgSO_4_, 0.1 mM EGTA), with the specific salt gradient reported in Supplemental Fig. S1*B*, along with a plot of three replicate chromatograms, monitored at 280 nm (protein), 260 nm (RNA/DNA), and 320 nm (aggregate) absorbance wavelengths. Fractions of 500 μl were collected into a 96-well plate, concentrated using a Sartorius Vivaspin 500 10,000 MWCO centrifugal concentrator, and subsequently analyzed by MS. The fractions selected had a final salt concentration of 500 to 525 mM NaCl. The protein concentration was quantified using a DC Bradford Assay (Bio-Rad) with a final protein concentration total yield of the pooled fractions of 1.0 to 1.3 mg/ml.

### Protein Mass Spectrometry

Proteins were identified and quantified using a Thermo Orbitrap Fusion Lumos tribrid mass spectrometer. Peptide separation was performed via reverse-phase chromatography on a Dionex Ultimate 3000 RSLCnano UHPLC system (Thermo Fisher Scientific), employing a C18 trap coupled to an Acclaim C18 PepMap RSLC column (Dionex; Thermo Fisher Scientific). Peptides were introduced by nano-electrospray for data-dependent tandem MS. Mass spectra were acquired using a standard top-speed HCD MS1-MS2 method, and the data were analyzed using the Proteome Discoverer standard workflow (Thermo Fisher Scientific).

Mass spectra files were analyzed using Proteome Discoverer 2.3. The spectra were searched against the complete 12,726 protein *D. discoideum* proteome obtained from UniProt in January 2024, as well as a 379 protein contamination database provided by the Hao group (30). Tryptic peptides were considered with a maximum of two missed cleavages. The analysis was performed with a minimum precursor mass of 350 Da and a maximum precursor mass of 6000 Da, applying a precursor mass tolerance of 10 ppm, and dynamic modifications considered on the protein N-terminus included methionine loss (−131.040 Da) and methionine loss with subsequent acetylation (−89.030 Da). FDR targets for peptide identification were set with a strict threshold of <1%. The FDR criterion for protein identification mirrored those for peptide identification, with a strict target of <1% and a relaxed target of <5%. The proteins listed in Supplemental Table S1 represent only those with high-confidence identifications, corresponding to an FDR of <1% and ≥2 unique peptides, with peptide identifications provided in the supporting tab of Supplemental Table S1.

### Cryo-EM Sample Preparation and Data Collection

Concentrated protein fractions from the mixed-bed IEX were adjusted to a final composition of 50 mM Hepes, pH 7.4, 100 mM NaCl, 3 mM MgSO_4_, 0.1 mM EGTA, 1 mM DTT, 0.1 mM PMSF, Roche cOmplete mini EDTA-free protease inhibitors, Roche PhosSTOP EASY phosphatase inhibitors, and 2% glycerol. This adjustment was made to lower the salt concentration and incorporate a cryoprotectant for subsequent plunge-freezing. A 3 μl aliquot of the concentrated protein solution was applied to a glow-discharged C-Flat Holey Thick Carbon 1.2/1.3 Cu400 grid, maintained at 100% humidity at 4 °C. Using an FEI Vitrobot Mark IV (Thermo Fisher Scientific), the grid was blotted for 10 s with a blot force of 1 and a wait time of 5 s, before being plunged into liquid ethane. A total of 332 micrographs were collected using a 200 kV FEI Glacios (Thermo Fisher Scientific) cryo-transmission electron microscope equipped with a Falcon IV direct electron detector (FEI). Exposures were collected with a calibrated pixel size of 0.933 Å/pixel, a total electron dose of 49 e−/Å^2^, and a defocus range between −1.5 and −2.5 μm.

### Cryo-EM Data Processing, Building, and Refinement

On-the-fly data processing was conducted using cryoSPARC Live (26) encompassing motion correction, defocus estimation, micrograph curation, and particle picking. Of the 332 micrographs acquired, 276 were retained for further analysis. The excluded micrographs were removed primarily due to excessive ice thickness and beam-induced drift, which compromised image quality and downstream interpretability. Subsequent data processing steps, including particle extraction, curation, and refinement, were performed using cryoSPARC v4.5 (31). During cryoSPARC Live processing, we employed a circular blob picker that was searching for particles between a maximum diameter of 270 Å and a minimum diameter of 200 Å, with an extraction box size of 512 pixels. The EM processing pipelines for the four reconstructed complexes are presented in full in Supplemental Figs. S2–S5 and are summarized in brief as follows. Map resolution and quality were assessed using the Validation (FSC) job in cryoSPARC, applying the gold-standard FSC criterion with a 0.143 threshold. Additional evaluation of map quality was performed using the *mtriage* tool in Phenix. Corresponding particle counts, FSC thresholds, and final resolution estimates are provided in Supplemental Table S2. Protein complex identification proceeded in parallel with cryo-EM density refinement, based on AlphaFold modeling of the top MS-identified proteins, comparing the predicted structures of proteins found experimentally to be present in the sample with the experimental cryo-EM densities, as detailed below.

For the 20S proteasome, particles from 2D classification were subjected to *ab initio* 3D classification. The best-resolved class was then refined by homogeneous refinement without imposed symmetry (C1), followed by a second homogeneous refinement with C2 symmetry. A final nonuniform refinement was performed with C2 symmetry maintained. Although higher symmetries (C7 and D7) were tested and yielded reconstructions with nominal resolutions exceeding those reported here, visual inspection revealed structural features inconsistent with previously published high-resolution proteasome structures. These discrepancies likely reflect symmetry-related artifacts amplified by the limited number of particles, rather than genuine improvements in structural accuracy. For this reason, the final reconstruction was carried out using C2 symmetry only. The final reconstruction was generated from a curated subset of 549 particles.

For the Odo2 complex, multiple rounds of 2D classification and *ab initio* reconstruction were carried out to refine the particle stack. Selected particles underwent an *ab initio* 3D reconstruction, followed by homogeneous refinement (C1). A subsequent homogeneous refinement with octahedral (O) symmetry was performed, and duplicate particles were removed prior to a final homogeneous refinement with O symmetry imposed. Alternate symmetry groups (C4 and C6) were also tested but resulted in lower resolution reconstructions. The application of O symmetry was supported by the distinct square-like features observed in representative 2D class averages and led to a substantial improvement in overall resolution. The final reconstruction was generated from a curated subset of 836 particles.

For the hexameric star complex, multiple rounds of 2D classification and *ab initio* 3D classification were used to refine the particle stack. Particles selected from 2D classes were subjected to *ab initio* 3D reconstruction; the best class then underwent homogeneous refinement without imposed symmetry (C1), followed by a final nonuniform refinement with no symmetry imposed (C1). Despite these iterative refinement steps, the resulting map remained poorly resolved, as indicated by fluctuations in the FSC curve (Supplemental Fig. S5*C*). The limited resolution and overall map reliability are likely attributable to pronounced preferential particle orientation, as demonstrated by the angular distribution plot (Supplemental Fig. S5*D*). Higher symmetries (C2 and C6) were tested and yielded reconstructions with no improvement to resolution, and the resulting reconstructions were accompanied by more prominent symmetry-related artifacts. The final reconstruction was generated from a curated subset of 2113 particles.

For Pks16, iterative 2D classification followed by *ab initio* 3D classification was used to refine the particle stack. Particles selected from 2D classes underwent *ab initio* 3D reconstruction; the best class was then subjected to homogeneous and nonuniform refinement with C2 symmetry imposed. The particle stack was further refined by removing duplicate particles, with final homogeneous and nonuniform refinements performed with C2 symmetry. Templates were generated from the volume obtained in the previous nonuniform refinement. A template picker was then employed to select particles, which underwent *ab initio* reconstruction to reduce potential for template-induced bias, followed by homogeneous and nonuniform refinement, again with C2 symmetry imposed. After a final round of removing duplicate particles, the dataset was subjected to homogeneous refinement, followed by a final nonuniform refinement, which produced the reconstruction used for model interpretation. The final reconstruction was generated from a curated subset of 5611 particles.

Note that for the case of Pks16, Guinier plot B-factors were low (28.6), suggesting that an increase in particle count would potentially provide additional resolution improvements. However, the remaining structures showed very high B-factors (>120), suggesting that small increases in particle counts would not contribute much to improving overall resolution. As the main focus of this paper is the Pks16 structure, we did not pursue improvements on the remaining structures.

### Model-Building and Refinement

Initial structural models were generated with AlphaFold 3 and visualized using UCSF ChimeraX (32). These models were further refined and validated using the phenix.real_space_refine tool in Phenix, which includes comprehensive MolProbity (33) validation metrics, as detailed next for each complex.

For the well-characterized 20S proteasome, the structure was readily identifiable by visual inspection, and MS confirmed the presence of all 14 subunits, supporting the model assignment. For Odo2 and Pks16, AlphaFold 3 models of the most abundant MS-identified proteins were evaluated, with attention to their potential multimeric assemblies. These models were fitted to the cryo-EM density using the *Fit-in-Map* and real-space correlation tools in ChimeraX to assess compatibility and refine positioning. For the case of Pks16, the KR_s_/MT region was independently fitted to density using ISOLDE (34), the model optimized by Namdinator, and the full model refined using Phenix, after substituting residues 2129 to 2165 and 2191 to 2215 from the ModelAngelo-built structure, as these helices were shifted by one helical turn in the AlphaFold 3 model relative to the cryo-EM density. For the case of Odo2, using AlphaFold 3, we evaluated the potential to form octahedral assemblies for all dehydrogenases in the sample and for the highest abundance proteins with compact folds, after eliminating coiled coil or unstructured proteins based on their predicted monomeric structures or proteins whose known oligomeric assemblies differed markedly from the observed octahedral density (*e.g.* as for the TRAPP and 3-methylcrotonyl-CoA carboxylase complexes). For the final unresolved hexameric star-shaped complex, each high-abundance protein identified by MS was modeled using AlphaFold 3, including multimeric predictions where applicable. These candidate models were systematically compared to the cryo-EM density using ChimeraX’s *Fit-in-Map* function. As described earlier, the density associated with this complex exhibited significant preferential particle orientation and poor resolution, as indicated by fluctuations in the FSC curve. These factors prevented confident structural assignment, and the identity of this complex remains undetermined.

### AlphaFold 3 Modeling of DdPKSs

As AlphaFold 3 generally correctly predicted the structure of Pks16, especially at the local domain level, we used it to explore potential interactions of the acyl carrier protein (ACP) domains (which was not resolved by cryo-EM for Pks16) with other domains in the predicted structures of the ketosynthase (KS)-ACP regions for the other 39 *Dd*PKSs (domains defined in Abbreviations). PKSs were split into two parts, as 4920 residues is the limit with four NADPH molecules. To preserve homodimeric interactions, KS+AT+DH and KS∗+DH+MT+KR_s_+ER+KR_c_+ACP dimers were independently predicted (KSATDH_PksXX.pdb and noAT_PksXX.pdb Data Files; KR_s_ and KR_c_ are the structural and catalytic subdomains of KR) and superposed through the KS dimer (∗ indicates 3 KS residues at Pks16 positions 86–88 were changed to aspartate to avoid the dominant KS/ACP association, Supplemental Fig. S8). As the merged models (MergedPksXX.pdb Data Files, native residue numbering) contain KS+AT from the first structure and DH+MT+KR_s_+ER+KR_c_+ACP from the second structure, they also contain a small defect at their junction, downstream of the conserved proline at position 935. NADPH is equivalently bound to each ketoreductase (KR) but predicted to bind through diverse modalities to enoylreductase (ER) and not predicted to bind Pks10 ER. The prediction for the unusual KS+AT+DH+KR_s_+ER+KR_c_+ACP portion of Pks37 was obtained by supplying AlphaFold 3 with two copies of residues 4 to 2503 from Pks37 (Pks37.pdb Data File).

To investigate the association of KS and ACP, two copies of KS+AT and ACP were supplied to AlphaFold 3 for each *Dd*PKS. A consensus association was identified (KSATACP_PksXX.pdb Data Files); it is similar to the KS/ACP association formed during the extension reaction in modular PKSs (35, 36). To investigate the association of acyltransferase (AT) and ACP, one copy of both the AT and ACP domain was supplied. From the 200 returned predictions (5 per job), a consensus docking site was identified (ATACP_PksXX.pdb Data Files); it is similar to the AT/ACP association in modular PKSs (37). To investigate the association of methyltransferase (MT) and ACP, a consensus docking site was identified from the KS∗+DH+MT+KR_s_+ER+KR_c_+ACP dimers, and more examples were obtained through supplying MT+KR+ACP monomers to AlphaFold 3 (MTACP_PksXX.pdb Data Files). To investigate the association of KR and ACP domains, a consensus docking site was identified from the KS∗+DH+MT+KR_s_+ER+KR_c_+ACP dimers, and more examples were obtained through supplying MT+KR+ACP monomers to AlphaFold 3 (KRACP_PksXX.pdb Data Files). To investigate the association of dehydratase (DH) and ACP domains, a consensus docking site was identified from the KS∗+DH+MT+KR_s_+ER+KR_c_+ACP dimers (DHACP_PksXX.pdb Data Files). No consensus docking site was observed from isolated DH and ACP domains. To investigate the association of ER and ACP domains, a consensus docking site was identified from the KS∗+DH+MT+KR_s_+ER+KR_c_+ACP dimers (ERACP_PksXX.pdb Data Files); it is similar but distinct from the ER/ACP association in modular PKSs (38). Structures containing consensus associations were superposed with corresponding Pks16 domain(s) for analysis (for KS/ACP, the KS dimer; for AT/ACP, the AT of chain B; for MT/ACP, the large MT subdomain of chain A; for KR/ACP, the KR_s_ of chain A; for DH/ACP, the DH of chain A; for ER/ACP, the ER of chain A). Each of the PksXX.pdb Data Files containing an AlphaFold 3 prediction is accompanied by a PksXX.cif Data File, a PksXX.json Data File containing the pLDDT, PAE, and other metrics that can be viewed in programs such as ChimeraX, and a PksXX_input.json Data File containing the input parameters.

## Results and Discussion

### Fractionation and Enrichment for High-Molecular-Weight Macromolecular Assemblies

**Fig. 1:**
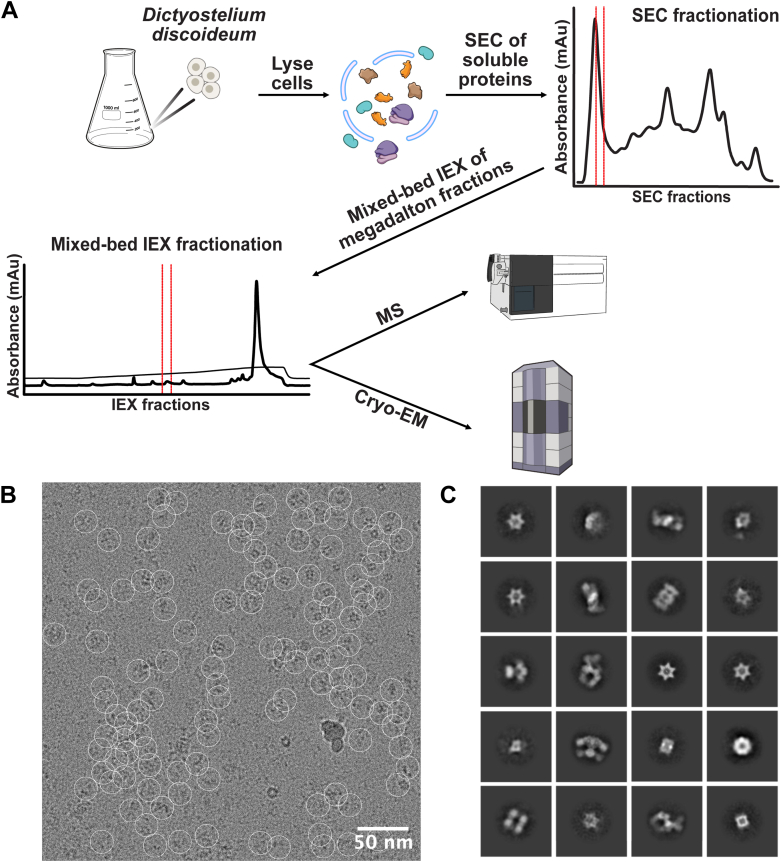
**Overview of a small pilot survey of high molecular weight complexes in *Dictyostelium discoideum.****A*, cells were collected during their vegetative stage and lysed using Igepal. The lysate was first fractionated using SEC chromatography and then by mixed-bed IEX to further reduce the heterogeneity of the protein mixture. Proteins enriched from SEC-IEX fractionation were loaded on cryo-EM grids and visualized, and in parallel, proteins were identified by MS. *B*, cryo-EM micrograph of proteins from the concentrated mixed-bed IEX fraction. Particles were picked using cryoSPARC live using template-free auto-picking (*white circles*). Scale bar represents 50 nm. *C*, 2D class averages for a subset of protein complexes with distinctive shapes. Class averages were computed using a 24 nm diameter circular mask. SEC, size-exclusion chromatography; MS, mass spectrometry; IEX, ion-exchange chromatography.

We sought to apply shotgun EM to survey high abundance high molecular weight protein complexes present in *Dictyostelium* slime mold cell lysate. As the complexity of unfractionated cell lysate is too high to solve individual structures, we first performed chromatography to reduce the complexity. In this study, we employed a rapid and efficient two-step biochemical fractionation of detergent-solubilized lysates from *D. discoideum* to isolate large macromolecular assemblies in a near-native state. Prior to fractionation, the protein assemblies were ultracentrifuged to prevent liposome formation in downstream processes. Native protein complexes were then separated according to molecular mass using SEC. SEC has become a widely adopted fractionation technique (7, 9), with the selection of high-molecular-weight fractions offering the advantage that larger proteins are readily observed upon visual inspection by transmission electron microscopy. Three high-molecular-weight fractions were collected from SEC and subjected to mixed-bed IEX to further separate the macromolecular assemblies based on surface charge properties. Three IEX fractions were then analyzed in parallel by MS for proteomic identification and by cryo-EM for structural characterization of the assemblies (Fig. 1*A*). As this was an initial pilot study to evaluate feasibility of this method for future scale-up, we have not yet exhaustively analyzed the remaining fractions. Our overall strategy was to compare the cryo-EM densities to predicted 3D structures of the most abundant MS-identified proteins, using either sequence-based structural homology (testing candidate PDB structures from sequence homologs) or full *de novo* structure prediction (AlphaFold 3).

In particular, cryo-EM enables the reconstruction of densities for macromolecular assemblies, but associating protein identities with these densities can be challenging without a comprehensive catalog. To facilitate this process, we employed MS in parallel, enhancing the identification and assignment of protein complexes to their corresponding EM densities. Following mixed-bed IEX, the fractions were subjected to trypsin digestion, and the resulting tryptic peptides were analyzed by liquid chromatography-tandem mass spectrometry. MS analysis identified 179 unique proteins, with 141 exhibiting six or more peptide spectral matches (PSMs) (Supplemental Table S1). This catalog of proteins captured a broad range of cellular and molecular functions, which guided and validated the identification and reconstruction of macroassemblies.

### Cryo-EM, 2D Classification, and Single-Particle Analysis of Enriched Complexes

To investigate the structural characteristics of the isolated protein complexes, cryo-EM was employed on the fractions obtained following IEX. A total of 332 micrographs were collected over a 3-h period, of which 276 were retained for subsequent processing based on image quality (see Experimental Procedures). Real-time processing was conducted using cryoSPARC Live, which included motion correction, defocus estimation, and particle selection. Template-free auto-picking facilitated the identification of over 37,000 particles (Fig. 1*B*). Subsequent 2D classification of these particles generated projections representing various orientations of the protein complexes, revealing several visually distinct structures (Fig. 1*C*). Notably, recognizable complexes such as the 20S proteasome, which is readily identifiable in complex mixtures (7, 8, 9), were observed.

A key challenge in cryo-EM analysis of heterogeneous mixtures is the identification of enriched protein complexes and the accurate assignment of corresponding densities to specific protein assemblies (14). By reducing sample complexity, we significantly improved the ability to match multiple 2D projections from the same complexes. The resulting 2D classes exhibit distinct features that enable effective sorting for further processing without exhaustive combination testing. For instance, several of the 2D classes display a hexameric star-like formation (Fig. 1*C*), which is clearly distinguishable from the well-characterized heptameric proteasome, allowing for the straightforward identification and grouping of all 2D projections exhibiting a size-matched hexameric arrangement.

After sorting the 2D classes, *de novo* reconstructions were generated and utilized in conjunction with the MS-derived identities, providing a foundation for subsequent refinement. Our general strategy was as follows: for well-characterized complexes such as the 20S proteasome, visual identification was straightforward given its distinctive, well-described structure across homologs, and assignments were independently corroborated by robust MS data. For all other cases, we evaluated AlphaFold 3–predicted structures of the most abundant proteins identified by MS, considering their potential multimeric assemblies, and assessed model compatibility with EM density using ChimeraX. The application of symmetry during reconstruction necessitates an understanding of the EM density to prevent the introduction of bias or artifacts that could undermine the resolution of the reconstructed structure. With the protein complexes identified through MS analysis, we were able to more accurately associate 2D projections with their corresponding protein assemblies and apply symmetry constraints appropriately, leading to improved structural models.

### Identification of Candidate Complexes by Comparing MS and Cryo-EM

**Fig. 2:**
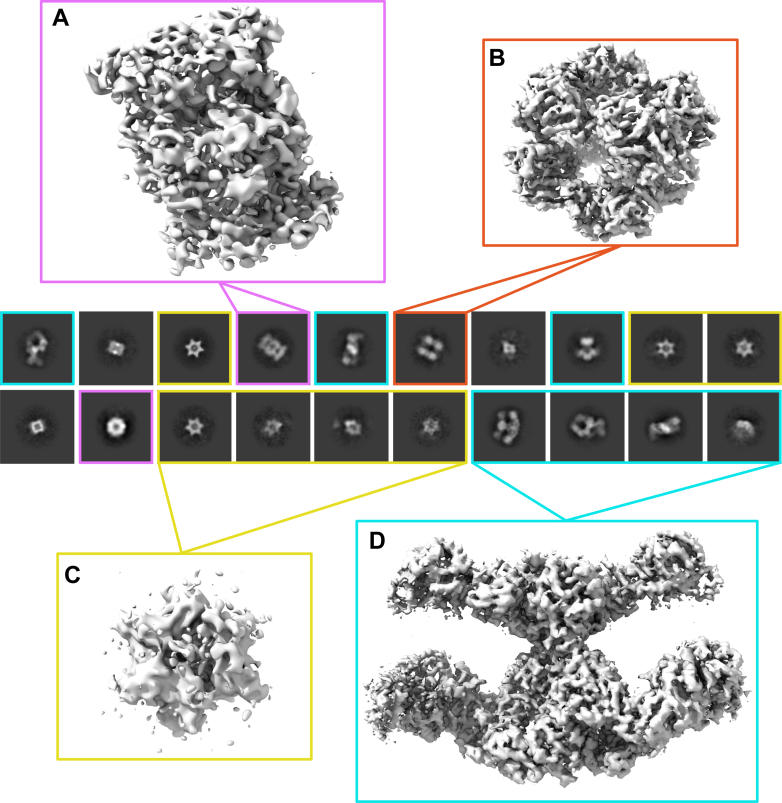
**Cryo-EM 2D-class average projections and electron densities of the major protein complexes observed.** Class average projections are outlined in colored squares by the corresponding protein complex: (*A*) 20S proteasome, *purple*; (*B*) dihydrolipoyllysine-residue succinyltransferase component of mitochondrial 2-oxoglutarate dehydrogenase complex, *orange*; (*C*) unidentified six-pointed star-shaped complex, *yellow*; and (*D*) polyketide synthase 16 (Pks16), *light blue*. Note that this was the complete set of “interesting” 2D classes obtained from this analysis.

One of the most prominent 2D classes identified was the proteasome, with MS data detecting all 14 subunits (7 α and 7 β subunits) of the 20S proteasome (Fig. 2*A*). Additionally, we observed a 2D class displaying a quadrilateral, box-like arrangement with four distinct corners, which corresponded to a 3D EM density exhibiting octahedral symmetry. Enforcing O symmetry brought the reconstruction from 12.04 Å (C1) to 5.72 Å (O) resolution, considerably improving the quality of the cryo-EM density map. Based on the cryo-EM density map and structural modeling of high-abundance proteins identified by MS utilizing AlphaFold 3 predictions, we could identify the protein as Odo2, the dihydrolipoyllysine-residue succinyltransferase subunit of the mitochondrial 2-oxoglutarate dehydrogenase complex, which forms a complex with octahedral symmetry and was present at high abundance in the MS data, with the 10th most PSMs among the proteins identified (Fig. 2*B* and Supplemental Table S1).

Among the other particles, we observed a distinct hexameric star-like structure that did not correspond to any of the high-abundance, well-characterized proteins in the sample (Fig. 2*C*). In contrast, we identified an extended structure that could be more confidently assigned as the most abundant protein in the sample: Pks16. This assignment was supported by both cryo-EM particle counts and MS data, with 5611 particles and 3222 total PSMs detected (Supplemental Tables S1, S2, Fig. 2*D*, and Supplemental Fig. S2). The number of particle counts and PSMs correspond to ∼15% and ∼26%, respectively, with a caveat that such percentages can be difficult to determine accurately due to missing observations and classification errors.

Although peptides were initially assigned to both Pks16 and the closely related Pks17—reflecting their 96% sequence identity—sequence alignment of all 87 detected peptides against the full-length Pks16 and Pks17 proteins revealed 29 peptides were uniquely matched to Pks16, while no peptides uniquely mapped to Pks17 (Supplemental Fig. S6). Thus, the detected peptides can be confidently attributed to Pks16, consistent with the fact that *Dictyostelium* was analyzed during its vegetative stage, when only Pks16 is transcriptionally active (25). Furthermore, using ModelAngelo, we conducted a HMMer search of the full *Dictyostelium* proteome to independently identify Pks16 as the most likely candidate sequence based on the cryo-EM map.

To identify the star-shaped macromolecular complex, we tested each high-abundance protein by modeling the protein with AlphaFold 3 (including considering multimeric assemblies) and comparing the models with the cryo-EM density. Two potential candidates emerged: (i) an ATP-dependent protease subunit HsLV protein with homology to known dodecameric *Escherichia coli* ATP-dependent protease subunit HsLV (PDB entry: 1E94) (39) or (ii) a partial assembly of the F1 ATP synthase. We first suspected the star might be a hexameric heat shock protein, and while hexameric heat shock protein HspA was highly abundant in the MS data, its predicted structure was a poor match for the cryo-EM density. In contrast, a dodecameric assembly of the ATP-dependent protease protein HsLV protein fitted the density well. However, our MS data suggested that it was only present at very low abundance across the biological replicate experiments, which considerably reduced our confidence in this identification.

**Fig. 3:**
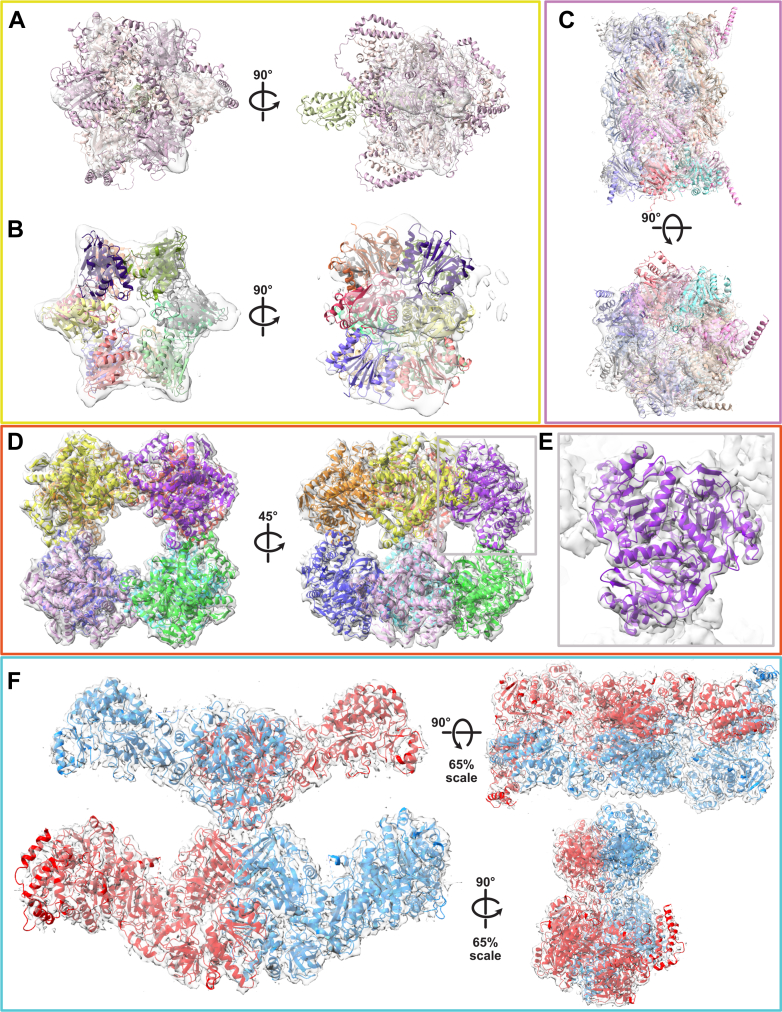
**Molecular models of reconstructed protein complexes.***A*, *Dictyostelium discoideum* ATP synthase model, predicted using AlphaFold 3, docked into the refined low-resolution map density of the hexameric star-like assembly. *B*, *D. discoideum* 12-mer structure of HsLV docked into the refined low-resolution map density of the hexameric star-like assembly. *C*, *D. discoideum* 20S proteasome model, predicted using AlphaFold 3, docked into the refined 9.52 Å resolution map density. *D*, *D. discoideum* Odo2 mitochondrial dihydrolipoyllysine-residue succinyltransferase subunit of the 2-oxoglutarate dehydrogenase complex, modeling residues 210 to 440 using AlphaFold 3, docked into the refined medium-resolution (5.72 Å) map density. *E*, magnified view of the Odo2 trimeric arrangement, corresponding to the region outlined in (*D*, charcoal-gray box). *F*, *D. discoideum* Pks16, modeled using AlphaFold 3, docked into the refined 3.94 Å resolution map density. Squares around each structure are colored to match those in Figure 2. PKS, polyketide synthase.

Thus, given the abundant visual representation of the hexameric structure in micrographs and particle counts from the 2D classes, we also considered ATP synthase as a potential candidate. ATP synthase was a plausible candidate, given its high abundance in the MS analysis and the reasonable alignment of the AlphaFold 3 model with the cryo-EM density. To this end, we used AlphaFold 3 to predict the structure of ATP synthase, incorporating three α-subunits, three β-subunits, and one γ-subunit to represent its natural assembly. The predicted model was then superimposed onto the low-resolution cryo-EM map density (Fig. 3*A*). Visualization of the model within the cryo-EM map supports ATP synthase as a strong candidate, as the predicted structure aligns with the EM density and retains key features, such as the density in the central cavity, where the γ-subunit is expected to reside. In contrast, the ATP-dependent protease subunit HslV protein complex, while exhibiting a well-defined hexameric structure when viewed down the central axis, does not account for the central density feature observed in the 2D classification, further distinguishing it from ATP synthase (Fig. 3*B*).

However, MS only identified the β-subunit of ATP synthase (specifically, the AtpB protein), with no evidence for either the α- or γ-subunits. While there is literature support for homo-hexameric β rings in other classes of ATP synthase homologs (*e.g.* AAA + ATPases (40), Rho (41)), they have not been documented for *D. discoideum* mitochondrial ATP synthase. Thus, we were not confident enough in either candidate (HsLV or AtpB) and this hexameric star structure remains unassigned; nonetheless, it is a very obvious feature among the slime mold lysate 2D projections that other researchers will certainly encounter in future studies.

### Modeling of Reconstructed Multiprotein Assemblies

In order to build models for each of the complexes, we initially computed candidate structures using AlphaFold 3 (Fig. 3, *A*–*F*), including symmetry as appropriate. For the well-characterized 20S proteasome, we maintained C7 symmetry when building the AlphaFold 3 model and fitted it into the 9.52 Å map (Fig. 3*C*). The dihydrolipoyllysine-residue succinyltransferase (E2) subunit of mitochondrial 2-oxoglutarate complex is previously known to form a homomeric 24-mer assembly exhibiting octahedral symmetry in *Homo sapiens* (42), so we modeled the *D. discoideum* Odo2 structure accordingly (Fig. 3*D*). We employed AlphaFold 3 to predict a trimeric structure of Odo2 (amino acids 210–439) and docked into the cryoEM density map using ChimeraX. Despite the medium resolution (5.72 Å) of the map, the predicted model aligned extremely well with the features of the EM density (Fig. 3*E*), especially in the sites of trimeric symmetry. Regions where AlphaFold 3 could not confidently predict structure (amino acids 1–209) were omitted, resulting in some voids in the density between the trimeric subunits, presumably occupied by the missing residues. Odo2 is known to interact in other species with the 2-oxoglutarate dehydrogenase (E1) subunit of the complex (43), encoded in *D. discoideum* by Odo1. We observed Odo1 in the MS data (as the 73rd most abundant protein), but at substoichiometric levels with Odo2, and we did not observe extensive unfilled density around the Odo2 octahedral core consistent with Odo1 presence.

Finally, we predicted the structure of the *D. discoideum* Pks16 protein using AlphaFold 3 (amino acids 1–2496) and observed a good agreement overall in the cryo-EM density, particularly at the level of individual domains, further improved by subsequent computational refinement of the model into the density (Fig. 3*F*). In general, the Pks16 density map exhibited higher resolution (<4.0 Å) in the core, while the outer arm and leg regions were of medium resolution (>6.0 Å), likely due to protein motion or the adoption of distinct conformations influenced by the presence of various molecules. Given the reduced resolution of these regions, we opted to use AlphaFold 3 for atomic model prediction rather than relying on alternative tools, such as ModelAngelo (44), for model generation directly in the cryo-EM density. The high consistency of the AlphaFold 3–predicted structure of the central body region, where ModelAngelo also generated a model, supported the use of AlphaFold 3 model for subsequent analyses and gave us confidence in constructing models for additional members of the *D. discoideum* PKS protein family (Supplemental Fig. S7).

### Modeling the Full Set of 40 *D. discoideum* PKSs

Pks16, thought to be the primary FAS of *D. discoideum* (Pks17 also operates during the late culmination stage) (25), is one of the 40 highly-related, iterative type I PKSs in this social amoeba (2, 4, 45). Based on our MS data, Pks16 was the sole PKS family member present in our fractions, providing an unambiguous identification, and was additionally confirmed as the top match between our experimental density and all possible *Dictyostelium* proteins using ModelAngelo. As noted above, while we experimentally determined its structure by cryo-EM, we obtained a reasonable model using the protein structure prediction tool AlphaFold 3, especially at the domain level, with some differences in relative domain orientation, as documented in Supplemental Fig. S7, and with the KR_s_/MT region a notable outlier, constrained by its lower quality EM map. Thus, a potential caveat of this approach is that while AlphaFold 3 can accurately model domains and identify domain-domain contact surfaces, the relative angular orientations of otherwise well-modeled individual domains may be less accurate.

**Fig. 4:**
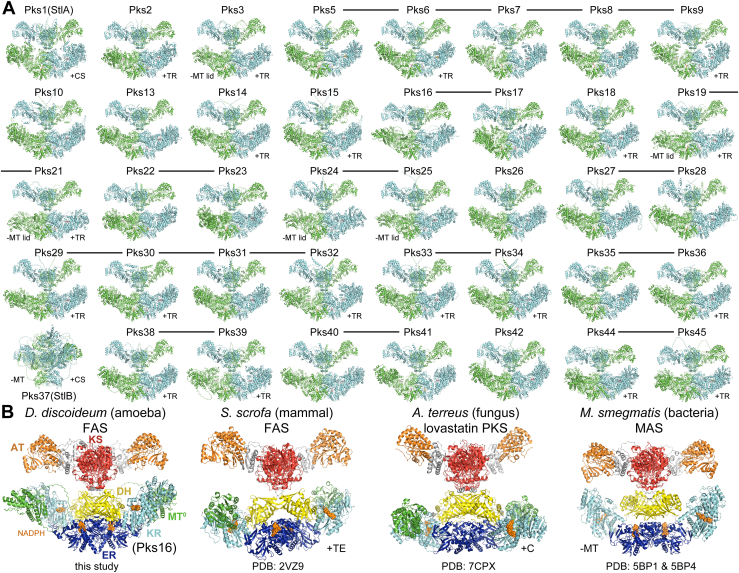
**Structural relationships between Pks16 and other dimeric synthases from *Dictyostelium discoideum* as well as mammalian, fungal, and bacterial species.***A*, Pks16 is one of the 40 *Dd*PKSs. With the exception of Pks37, the KS-ACP architectures predicted by AlphaFold 3 are highly similar (Merged_PksXX.pdb and Pks37.pdb Data Files). Synthases whose genes are chromosomally adjacent and highly homologous are connected by lines (4). Those possessing a C-terminal chalcone synthase (CS) or thioester reductase (TR) domain are indicated, as are those lacking an MT domain or MT lid (positions 1254–1414 and 1575–1594). *B*, the principal *D. discoideum* FAS, Pks16, is compared with the porcine FAS, the lovastatin PKS, and MAS. The porcine FAS and lovastatin PKS possess a C-terminal thioesterase (TE) and condensation (C) domain, respectively. MAS does not contain an MT domain. PDB codes are indicated. AlphaFold 3 models for Pks16 and MAS are shown. KS, ketosynthase; AT, acyltransferase; DH, dehydratase; MT, methyltransferase; MT^0^, inactive MT; ER, enoylreductase; KR, ketoreductase; ACP acyl carrier protein; TE, thioesterase; C, condensation domain; PKS, polyketide synthase; FAS, fatty acid synthase; MAS, mycocerosic acid synthase.

Since AlphaFold 3 cannot predict structures larger than 5000 residues, to better model dimeric interfaces, the 5206-residue Pks16 homodimer was predicted as two dimeric fragments and merged by superposing their KS domains (see Experimental Procedures). Similar structures for 38 of the other PKSs were equivalently generated using AlphaFold 3 (Merged_PksXX.pdb data files, Fig. 4*A* and Supplemental Fig. S5). Because the functions of only a few of these PKSs are known, this represents a plethora of structural data. From the 40 PKS structures, one can only hypothesize how Pks1 (also referred to as StlA) yields the short acyl chain incorporated into differentiation factor DIF-1 (46), how Pks5 yields the spore germination suppressing dictyodenes (5), and how Pks16 yields fatty acids (4) as the polyketides generated by the other PKSs have not been identified. Much can be learned about how the 40 *Dd*PKSs operate through a comparative analysis of their structures.

The ACP domain plays a prominent role in these iterative type I PKSs, repeatedly docking to each of the other domains to perform the rounds of elongation and processing that yield the product(s) of a synthase (45). In the *Dd*PKSs, the other domains include an AT that transfers a malonyl extender unit from malonyl-CoA to the ∼18 Å-phosphopantetheinyl arm of ACP as well as a KS that acquires a processed intermediate from an acyl-ACP and fuses it with malonyl-ACP through a decarboxylative Claisen-like condensation to generate a β-ketoacyl–ACP intermediate. Processing domains may also be present, such as an MT that transfers a methyl group from SAM to the α-carbon of the β-ketoacyl–ACP intermediate, a KR that can set stereochemistries at the α- and β-positions as it uses NADPH to generate a β-hydroxyacyl–ACP intermediate, a DH that eliminates water to generate an α/β-enoyl–ACP intermediate, an ER that can set stereochemistry at the α-position as it uses NADPH to reduce the α/β-enoyl–ACP intermediate, and an offloading enzyme that releases the product(s) from the synthase. While a processing enzyme may be present in a synthase, it may not be functional. Likewise, while a processing enzyme may be functional, it may not operate every round. In Pks5, DH does not work on intermediates smaller than a triketide, and MT does not work on intermediates smaller than a heptaketide. Processing enzymes may also perform different chemistry on different acyl-ACP intermediates, as in the hypothemycin PKS where KR generates an L-oriented hydroxyl group when reducing the diketide intermediate and a D-oriented hydroxyl group when reducing longer intermediates (47). Thus, the detailed interactions between acyl-ACPs and other domains within a PKS control the product(s) it generates.

### Synthase Architecture

The iterative type I synthases responsible for biosynthesizing fatty acids for an organism (FASs) usually possess one of two very different domain organizations (48). A hexameric architecture is present in the FASs of fungi and mycolic acid–producing bacteria, whereas a dimeric architecture is present in the FASs of nonfungal, nonplant eukaryotes. While fungi and mycolic acid–producing bacteria employ hexameric synthases to generate fatty acids, they also utilize dimeric synthases to generate diverse non-fatty acid polyketides. As examples, the fungus *Aspergillus terreus* and the mycolic acid–producing *Mycobacterium smegmatis* respectively produce lovastatin and mycocerosic acid using PKSs with architectures equivalent to dimeric FASs. Organisms with dimeric FASs do not usually possess additional iterative type I PKSs; however, social amoeba are a marked exception. Distinguishing their FAS from their PKSs based on sequence is not trivial, and the structural information reported here does not aid much either, since Pks16 superposes well with 38 of the other 39 PKSs.

Pks16 can be compared to the few other dimeric synthases whose structures have been experimentally determined—the FAS from the mammal *Sus scrofa* (pig) (PDB: 2VZ9) (49), the lovastatin PKS from the fungus *A. terreus* (PDB: 7CPX) (50), and the mycocerosic acid synthase (MAS) from the bacterial species *M. smegmatis* (PDBs: 5BP1 & 5BP4) (Fig. 4*B*) (51). In each of the synthases, monomeric AT domains are rigidly connected to a KS dimer through a flanking subdomain (52), and monomeric KR domains are connected to DH and ER dimers that make contact with one another on the twofold axis. With the exception of MAS, a monomeric MT domain inserts into a loop of the KR structural subdomain, KR_s_. Whereas the KS+AT region is rigidly connected to the DH dimer in Pks16 and the lovastatin PKS, it is flexibly connected in the porcine FAS and MAS. ACP domains are not observed in any of these structures nor are the monomeric offloading enzymes of the porcine FAS or the lovastatin synthase [a thioesterase (TE) and condensation (C) domain, respectively] (53).

In the *Dd*PKSs, the tight interaction of three regions (positions 53–59, 926–937, and 959–962; position numbering is in reference to Pks16, Supplemental Fig. S9*A*) unite the KS and DH domains (NoAT_PksXX.pdb Data Files). A major contact is made by a highly conserved proline at position 935 that inserts into a pocket formed by residues at positions 53, 55, 56, and 59. Another is from the side chain of a residue at position 937 (most commonly isoleucine) inserting into a pocket formed by residues 55, 58, and 59. Another is from the side chain of the residue at position 59 (most commonly a leucine) inserting into a pocket made by residues at positions 935 to 937 and 959 to 962. These interactions ensure that the PKS monomers in the X-shaped dimer are linearly organized. The KS/DH connection in the lovastatin PKS is similar yet distinct. Its interface is larger and proportionally more contact is made at its center.

In the *Dd*PKSs, the DH/KR interface is primarily mediated by complementary interactions between residues in the last β-strand of the DH β-sheet (positions 1073–1076) and a KR_c_ helix (positions 2272–2283) (NoAT_PksXX.pdb Data Files) (Supplemental Fig. S9*B*). Most commonly, a phenylalanine at position 1073 inserts into a pocket formed by residues in positions 2276, 2279, 2280, and 2283, a threonine in position 1074 inserts into a pocket formed by 2272, 2275, and 2276, and a tryptophan at position 2272 contacts a leucine at position 1076. The NH_2_ of a conserved asparagine at position 2279 usually forms a hydrogen bond with the backbone carbonyl of the residue at position 1073. This interface is distinct from the DH/KR interfaces of the porcine FAS and the lovastatin PKS but most similar to the porcine FAS. The DH and KR domains of MAS do not share an interface.

While AlphaFold 3 performed exceedingly well to predict common architectures of the KS–ACP regions for 39 *Dd*PKSs, it has notable limits. Some, but not all, of the structures for Pks37 (also referred to as StlB) show its ER dimer on the opposite side of the KS dimer from the DH dimer. Perhaps the multiple sequence alignment used by AlphaFold 3 needs to contain more Pks37 homologs for more confident prediction. AlphaFold 3 seems not to be able to place the offloading enzymes present downstream of ACP in 21 of the *Dd*PKSs. Two of these (from Pks1 and Pks37) are dimeric chalcone synthases (PDB: 2H84) (46) likely located on the two-fold axis of the synthase, whereas the others are most likely monomeric thioester reductases. In hexameric FASs, the ACP domain is constrained by anchored, N- and C-terminal linkers. Thus, the C-terminal offloading enzymes of the *Dd*PKSs might be expected to share an interface with one of the PKS domains. However, for AlphaFold 3 to predict these putative interfaces may also require more sequence information from homologs.

### Elucidating the Reaction Chambers of the DdPKSs

The region within which the ACP domain diffuses to dock each of its cognate enzymes is known as the reaction chamber. The best characterized reaction chamber is that of the hexameric *S. cerevisiae* FAS, which has been observed through cryo-EM with ACP bound to each of its cognate enzymes (54). This ACP only needs to rotate and translate within a small volume to complete a reaction cycle since its docking sites are oriented towards the center of the reaction chamber.

While it should be possible to push cryo-EM studies of Pks16 to experimentally characterize the reaction chamber of a dimeric synthase, AlphaFold 3 is already able to make atomic-level docking predictions with high confidence. It predicts how ACPs dock with downstream KSs in modular PKSs, as corroborated by mutagenesis and cryo-EM studies (55, 56). It also predicts how ACPs dock with KRs (57), DHs (58), and ERs (38) in modular PKSs, as corroborated by mutagenesis studies. In the AlphaFold 3–predicted structures of the KS–ACP regions of the 40 *Dd*PKSs, ACP domains are frequently associated with cognate enzymes. Remarkably, these interfaces are similar to those experimentally observed or predicted for modular PKSs.

As some associations dominated others, we tested various strategies to obtain more examples of less frequently observed associations. For example, because ACP/KS associations are common in the KS+DH+MT+KR_s_+ER+KR_c_+ACP dimers, 3 KS residues at the predicted ACP/KS interface (positions 86–88) were changed to aspartates. Abrogating the ACP/KS interface rather than the KS domain was preferred since KS and DH share a large interface. ACP/ER associations dominated the resulting KS∗+DH+MT+KR_s_+ER+KR_c_+ACP dimers. To obtain more structures with ACP/MT and ACP/KR associations, we asked AlphaFold 3 to predict monomeric MT+KR+ACP structures.

**Fig. 5:**
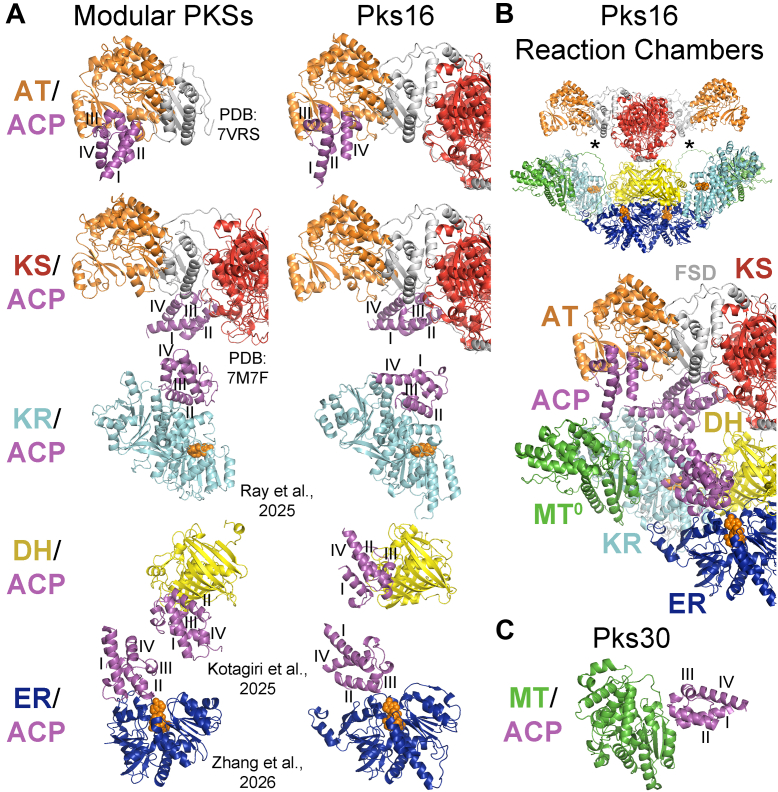
**ACP docking sites predicted by AlphaFold 3 define the Pks16 reaction chamber.***A*, the associations predicted for Pks16 were similar to the consensus associations for the 39 other *Dd*PKSs (the DH/ACP association is from Pks17, 96% identical to Pks16). They are similar to associations characterized in modular PKSs, the biggest exception being DH/ACP, where ACP cannot equivalently dock due to a steric clash that would result with ER. ACP helices I-IV are labeled. *B*, each Pks16 homodimer contains two equivalent reaction chambers (∗). An overlay of docking poses maps out the reaction chamber of Pks16 and the trajectory its ACP follows during a reaction cycle (see Supplemental Movie S1). *C*, an association predicted for Pks30 is representative of the MT/ACP consensus association observed for *Dd*PKSs. PKS, polyketide synthase; ACP, acyl carrier protein; MT, methyltransferase; ER, enoylreductase; DH, dehydratase.

One of the most frequent associations observed in the predictions of KS+DH+MT+KR_s_+ER+KR_c_+ACP dimers is between the KS and ACP domains (Fig. 5*A* and Supplemental Fig. S8*A*). To more completely investigate these interactions, AlphaFold 3 was asked to predict how the KS+AT dimer associates with two copies of ACP in each of the 40 *Dd*PKSs (KSATACP_PksXX data files). Not only were each of the predicted structures similar to one another, they were similar to both the cryo-EM structures reported from modular PKSs in which KS associates with a downstream ACP during the extension reaction (PDBs: 7M7F, 7S6C) (35, 36). While the KSs of modular PKSs possess a second ACP docking site employed during the transacylation reaction, it is thought that a single ACP docking site is employed in both the extension and transacylation reactions conducted by iterative synthases (55, 56). Thus, ACP visits this site at least twice per the reaction cycle, once to collect an acyl chain bound to the KS reactive cysteine through the extension reaction and once to transfer the extended and processed chain back to the KS reactive cysteine. Similar to the KS/ACP interface of modular PKSs, KS makes contact with the phosphopantetheinylated end of ACP at positions 81 (usually leucine) and 84–88. Unlike the interface of modular PKSs, a loop connecting flanking subdomain and AT (positions 534–541) also makes contact with ACP residues (downstream of helix I at positions 2528–2531; this interface is similar but different for Pks1 and Pks37). In Pks2, Pks22, Pks23, Pks44, and Pks45, AT residues also make contact with ACP residues (in helices I and IV).

The interface observed between AT and ACP in a modular PKS (PDB: 7VRS) is relatively small (Supplemental Fig. S10*B*) (37). Perhaps the phosphopantetheinyl arm significantly contributes to the AT/ACP binding interaction, as this moiety comprises the majority of the other AT substrate, malonyl-CoA. When AlphaFold 3 was asked to predict how ATs of the *Dd*PKSs associate with ACPs, consensus solutions similar to the modular PKS AT/ACP structure were obtained for 18 PKSs (ATACP_PksXX data files). The residues in AT positions 733 (usually phenylalanine) and 735 generally insert into pockets formed by residues in ACP positions 2543 (the phosphopantetheinylated serine), 2547, 2550, and 2564. An AT residue at position 835 (usually phenylalanine) generally inserts into a pocket between ACP helices I and II.

While only three PKSs showed an MT/ACP association in the KS∗+DH+MT+KR_s_+ER+KR_c_+ACP predictions, 15 more PKSs showed this consensus association in the MT+KR+ACP predictions (MTACP_PksXX.pdb Data Files) (Supplemental Fig. S10*C*). To our knowledge, MT/ACP interfaces for iterative type I PKSs have neither been characterized nor proposed in the literature. The predictions show MT associating with the phosphopantetheinylated end of ACP. The N-terminal residues of an MT helix (Pks30 positions 1508–1516; Pks30 is used as a reference for MT/ACP interactions since Pks16 does not have an active MT) make contact with the C-terminal residues of ACP helices I and II (Pks30 positions 2550–2551 and 2567–2575). A conserved serine (Pks30 position 1508) forms a hydrogen bond with the DSL aspartate (Pks30 position 2568) of ACP.

Eleven PKSs showed a KR/ACP association in the KS∗+DH+MT+KR_s_+ER+KR_c_+ACP predictions, and 10 more PKSs showed this consensus association in the MT+KR+ACP predictions (KRACP_PksXX.pdb data files) (Supplemental Fig. S10*D*). The interfaces are broadly similar to one another as well as to the interface predicted for B-type KRs from modular PKSs (57). The location of the phosphopantetheinyl arm attached to the conserved ACP serine (position 2543) affects how polyketides enter the KR active site and thus the stereochemical outcome of the reduction reaction. The predictions show how interactions between residues in ACP helices II and III (positions 2542–2569) and residues near the KR active site (positions 1782–1784, 2335, 2385–2386, 2428–2438, and 2478–2481) dictate the placement of the phosphopantetheinyl arm. Generally, the greater interaction between KR residues and ACP helix III residues leads to a rotation of ACP relative to how it is bound in modular PKSs.

The associations of DH and ER with ACP are apparently interconnected, with ER residues aiding the DH/ACP association and DH residues aiding the ER/ACP association (Supplemental Fig. S10*E*). DH may be more reliant on ER, as four PKSs in the KS∗+DH+MT+KR_s_+ER+KR_c_+ACP predictions show the DH/ACP consensus association compared to 27 PKSs that show the ER/ACP consensus association (DHACP_PksXX.pdb and ERACP_PksXX.pdb data files). When AlphaFold 3 is asked to predict DH/ACP associations in the absence of other domains, no consensus docking is observed. The DH and ACP domains cannot associate as predicted for modular PKSs due to steric clashes with the ER dimer (ERs in modular PKS are almost exclusively monomeric) (58, 59). In the consensus association observed for the *Dd*PKSs, ACP is relatively rotated 180°, similar to how AlphaFold 3 predicts ACP docks with DH in the porcine FAS and MAS. The ER residues that aid in the DH/ACP association (ER residues in positions 1857, 1890–1893, and 2175 contact ACP residues in positions 2524–2539, C-terminal to helix I) also aid in the ER/ACP association. Residues close to the DH active site (positions 1003–1004, 1043, 1149, and 1210) only make a few contacts with residues near the phosphopantetheinylated serine (position 2543) on ACP helices II and III.

The ER/ACP associations observed from 27 PKSs in the KS∗+DH+MT+KR_s_+ER+KR_c_+ACP predictions are consistent (Supplemental Fig. S10*F*). ER residues (positions 1856–1857, 1859, 1890–1893, 2171, and 2175) make contact with ACP residues in and between helices II and III (positions 2543–2565). At the center of these interactions is contact between two residues at the C-terminal end of an ER helix (positions 1890–1891) and an ACP side chain at position 2564 (predominantly isoleucine). These ER residues usually also make contact with an ACP valine conserved in position 2547. The side chains of ER residues at positions 1857, 1859, and 2171 (most often asparagine, glutamate, and lysine, respectively) usually form a hydrogen bond network with an ACP asparagine conserved in position 2560. A β-hairpin motif in DH [DxK(S/T)NEWI, not present in Pks16 or Pks17, but comprised of residues equivalent to those at positions 1035–1043] and neighboring DH residues make highly complementary interactions with residues between ACP helices I and II (positions 2529–2538). These contacts include a hydrogen bond between the aspartate from the DH β-hairpin motif and a conserved ACP asparagine (position 2531), a hydrophobic interaction between the isoleucine of the DH β-hairpin motif and a conserved ACP leucine (position 2534), a hydrogen bond between a DH serine/threonine with a semiconserved ACP aspartate (position 2538), and a salt bridge between a DH lysine and a conserved ACP aspartate (position 2533). The ACP/ER association is similar to, but distinct from, the association AlphaFold 3 predicts for the porcine FAS, MAS, and modular PKSs (38).

From the KS/ACP, AT/ACP, KR/ACP, DH/ACP, and ER/ACP associations predicted for Pks16, a movie was generated showing how ACP moves during a reaction cycle in the synthesis of a fatty acid (Supplemental Movie S1). ACP does not need to translate much in its reaction chamber (Fig. 5*B*). The reaction chambers of the other *Dd*PKSs may slightly differ due to the presence of C-terminal domains that further constrain ACP movement or functional MTs with which ACP must associate (Fig. 5*C*). However, given the structural relationships between Pks16 and other dimeric FASs/PKSs in amoeba, mammals, fungi, and bacteria, these molecular machines are likely to operate through similar dynamics.

## Conclusions

In this pilot study, we employed shotgun EM to analyze *D. discoideum* cell lysates, successfully isolating several of its high-molecular-weight multiprotein assemblies. By employing this methodology with heterogeneous mixtures, we were able to identify and reconstruct several protein complexes, including an octahedral assembly of the dihydrolipoyllysine-residue succinyltransferase subunit of the mitochondrial 2-oxoglutarate dehydrogenase complex, the 20S proteasome, and an unidentified hexameric star-like protein complex. Most notably, we serendipitously obtained a 3.9 Å experimental structure for a *D. discoideum* PKS, Pks16, the first of 40 *D. discoideum* PKSs to have an experimentally determined structure, which allowed us to perform a comparative analysis across the members of this important protein family and computationally characterize the reaction chambers of this important class of proteins.

## Data Availability

Mass spectrometry proteomics data was deposited in the MassIVE/ProteomeXchange database (60) under accession number PXD061189/MSV000097213. Cryo-electron microscopy data was deposited in the Electron Microscopy Data Bank (61) under accession numbers EMD-49490 (Odo2), EMD-49491 (Pks16), EMD-49492 (Hexameric star complex), EMD-49493 (20S proteasome). Coordinates for Pks16 and Odo2 were deposited in the Protein Data Bank as PDB 9NJU and 9NJT, respectively. Coordinates for all computationally predicted structures, including the 40 PKS proteins and modeled subsets of domains, are available through a Zenodo repository (10.5281/zenodo.17162530).

## Supplemental data

This article contains supplemental data.

## Conflicts of interest

The authors declare that they have no conflicts of interest with the contents of this article.

## Supplementary Data

Supplemental Figures and Legends

Hoogerbrugge Movie 1
