# Supplementary material for: Serendipity and the Slime Mold: A Visual Survey of High-Molecular-Weight Protein Assemblies Reveals the Structure of the Polyketide Synthase Pks16
Source: Mol Cell Proteomics. 2025 Dec 9;25(1):101484. doi: 10.1016/j.mcpro.2025.101484 (PMC12828403; doi:10.1016/j.mcpro.2025.101484)
Supplement: Supplemental Figures and Legends [file mmc3.docx]

**Supplemental Figures and Legends**

**Figure S1. Size exclusion and mixed bed ion exchange chromatograms.** (A) Size exclusion chromatography (SEC) traces of 3 biological replicate analyses of *D. discoideum* lysate overlaid with molecular weight standards, plotted in comparison with chromatograms from blue dextran (2 MDa) and a protein standard mix composed of thyroglobulin (669 kDa), apoferritin (443 kDa), alcohol dehydrogenase (150 kDa), and bovine serum albumin (66 kDa). Absorbance was monitored at 280 nm and was plotted following min/max normalization. The grey box indicates fractions collected for IEX separation. (B) Mixed-bed ion exchange (IEX) chromatograms of concentrated SEC fractions 47-52, with absorbance monitored at 280 nm (proteins), 320 nm (aggregates), and 260 nm (RNA/DNA), plotted without normalization. The grey box indicates fractions (46-49) collected for cryo-EM analysis. For reference, ribosomes elute in the SEC column in the gray box fractions, but shift differentially to the high salt wash (> 65 min) in the IEX column.

**Figures S2-5. Cryo-EM processing pipelines for (S2) polyketide synthase Pks16, (S3) the octahedral Odo2 dihydrolipoyllysine-residue succinyltransferase complex, (S4) the 20S proteasome, and (S5) the hexameric star complex.** In each case, the specific cryo-EM workflow is presented with a view of the final EM density along with the statistics of the final reconstruction. (A) Data processing and refinement pipeline for the complex using cryoSPARC v4.5. The symmetry utilized at each refinement step is noted at the bottom right hand corner of the respective refinement. (B) Cryo-EM volume colored by local resolution estimation (units in Å). (C) Gold-standard Fourier shell correlation (GSFSC) curve using an FSC threshold of 0.143, as calculated by cryoSPARC. (D) Viewing angle distribution plot.

**Figure S6. Mass spectrometry-identified peptides for Pks16.** 87 unique Pks16 peptides were identified by mass spectrometry and are underlined in the *D. discoideum* Pks16 sequence.

**Figure S7. Comparisons between ModelAngelo-built model, AlphaFold model, and final refined model of Pks16.** (A) Model created by ModelAngelo. (B) Model created by AlphaFold 3, colored based on root mean square deviation (r.m.s.d) of alpha carbons to the corresponding residues in the ModelAngelo model, computed by aligning with ChimeraX matchmaker and calculating sequence RMSD to the ModelAngelo model. Colors range from blue (<0.5 Å r.m.s.d.) to red (>1.5 Å r.m.s.d.); gray residues were absent from the ModelAngelo model. (C) Final, refined Pks16 model, colored based on root mean square deviation (r.m.s.d) of alpha carbons to the corresponding residues in the ModelAngelo model, computed and colored as in (B). (D) Final refined Pks16 model, color based on root mean square deviation (r.m.s.d) of alpha carbons to the model created by AlphaFold 3 when fit on a whole protein level. Colors range from blue (<0.5 Å r.m.s.d.) to red (>3.0 Å r.m.s.d.). (E) Final refined Pks16 model, color based on root mean square deviation (r.m.s.d) of alpha carbons to the model created by AlphaFold 3 when fit on a domain-by-domain basis. Colors range from blue (<0.5 Å r.m.s.d.) to red (>3.0 Å r.m.s.d.). (F) Table containing values of root mean square deviation (r.m.s.d) of alpha carbons from (D) and (E) between domains.

**Figure S8. Multiple sequence alignment for the KS-ACP regions of the *Dictyostelium discoideum* PKSs (except Pks37).** Domain boundaries are indicated with colored bars. Numbering and secondary structure is based on Pks16. “D” indicates the 3 KS positions (residues 86-88) at which aspartate was substituted to abrogate the association of KS and ACP in AlphaFold predictions.

**Figure S9. Structural interfaces between Pks16 domains.** (A) A stereodiagram shows that the Pks16 KS/DH interface is rigid, similar to that of the lovastatin PKS. In contrast, the KS and DH domains of the porcine FAS are only tethered by unstructured linkers. (B) The DH/KR interface is distinct from those of the lovastatin PKS and the porcine FAS.

**Figure S10. ACP docking sites.** Stereodiagrams show how the Pks16 ACP associates with its cognate enzymes. Also shown is a representative MT/ACP association from Pks30, as Pks16 does not have an active MT. The phosphopantetheinylated ACP serine is indicated with “**S**”. **(**A) KS/ACP (the AT loop shown is equivalent to the loop that makes contact with ACP in Pks2, Pks22, Pks23, Pks44, and Pks45). (B) AT/ACP. (C) MT/ACP (Pks30 numbering). (D) KR/ACP. (E) DH/ACP (the solution from Pks17, nearly identical to Pks16, is shown with Pks16 numbering). (F) ER/ACP (* indicates where a DH β-hairpin is located in most *Dd*PKSs that contacts ER-docked ACPs.

**
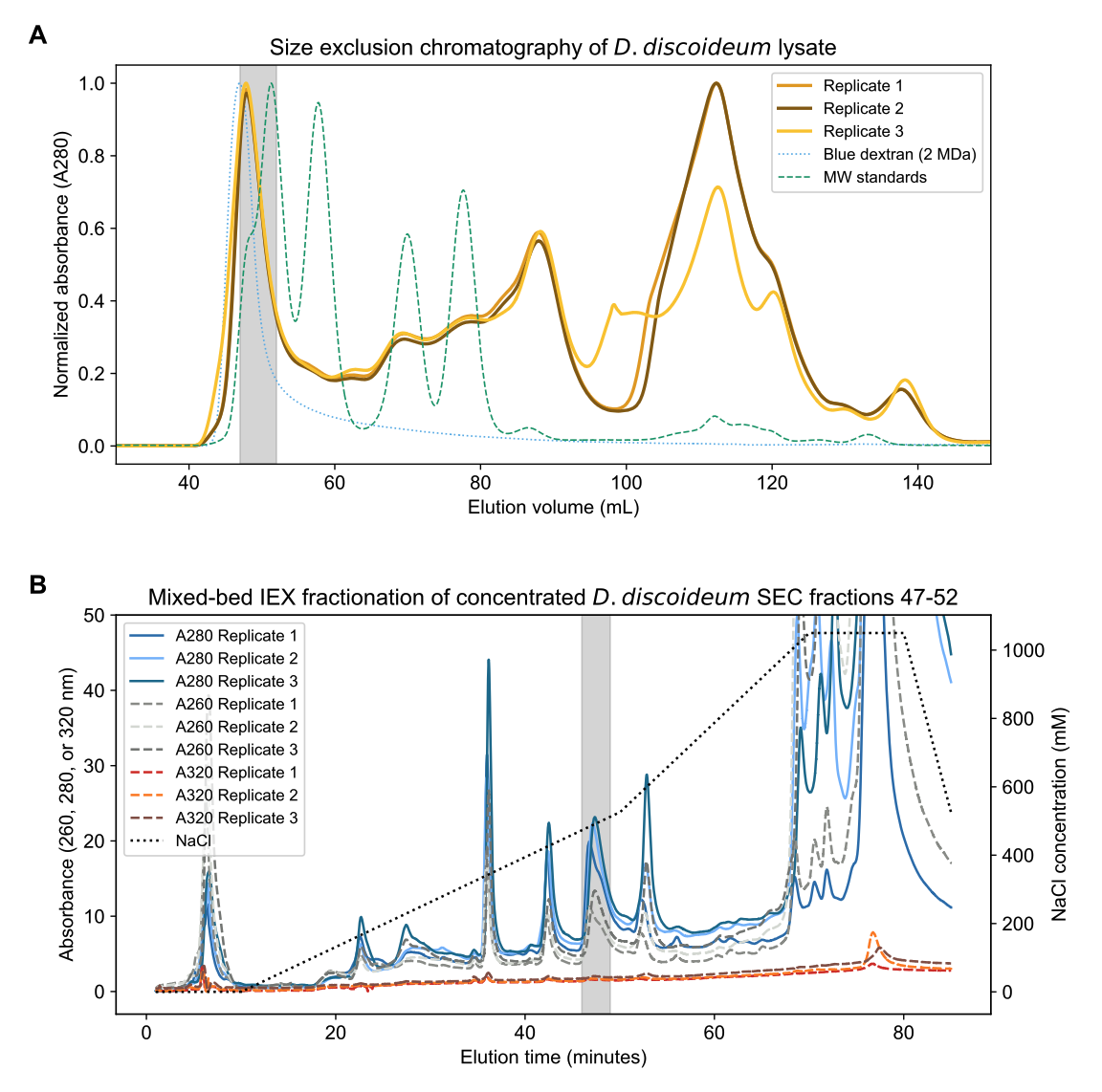
**

**Figure S1. Size exclusion and mixed bed ion exchange chromatograms.**

**
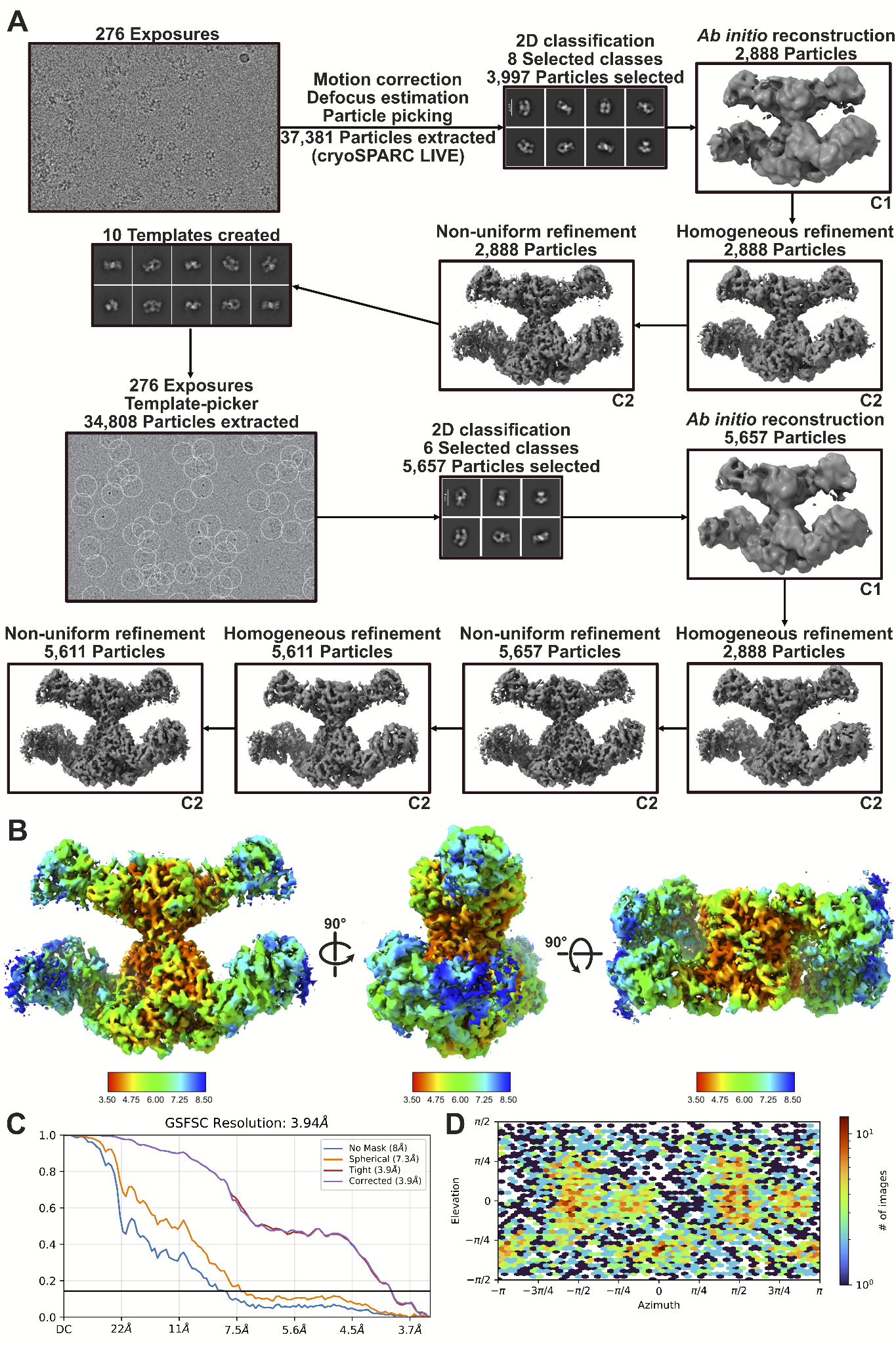
**

**Figure S2. Cryo-EM processing pipeline for the *D. discoideum* polyketide synthase Pks16.**

**
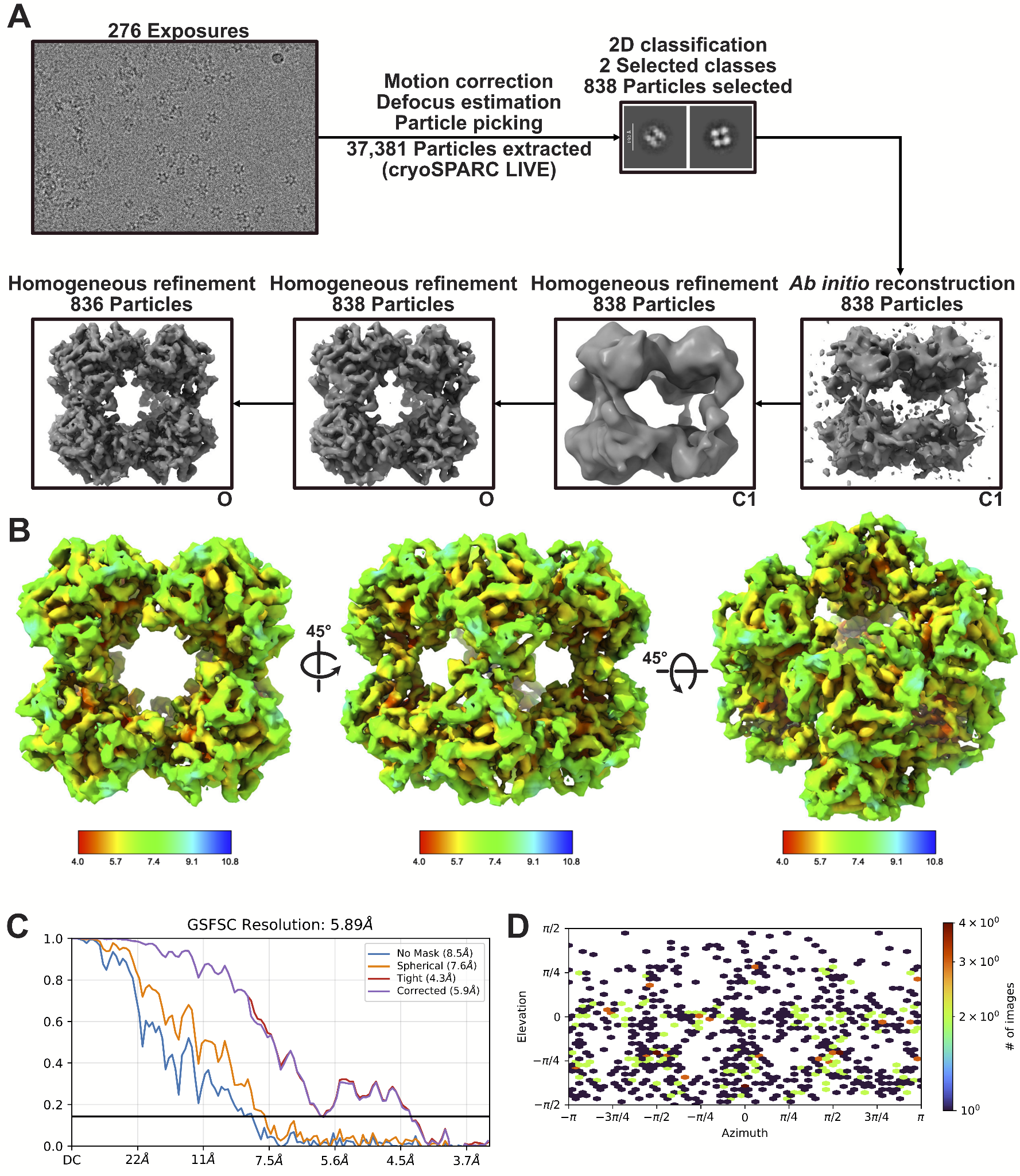
**

**Figure S3. Cryo-EM processing pipeline for the octohedral assembly of the *D. discoideum* Odo2 dihydrolipoyllysine-residue succinyltransferase.**

**
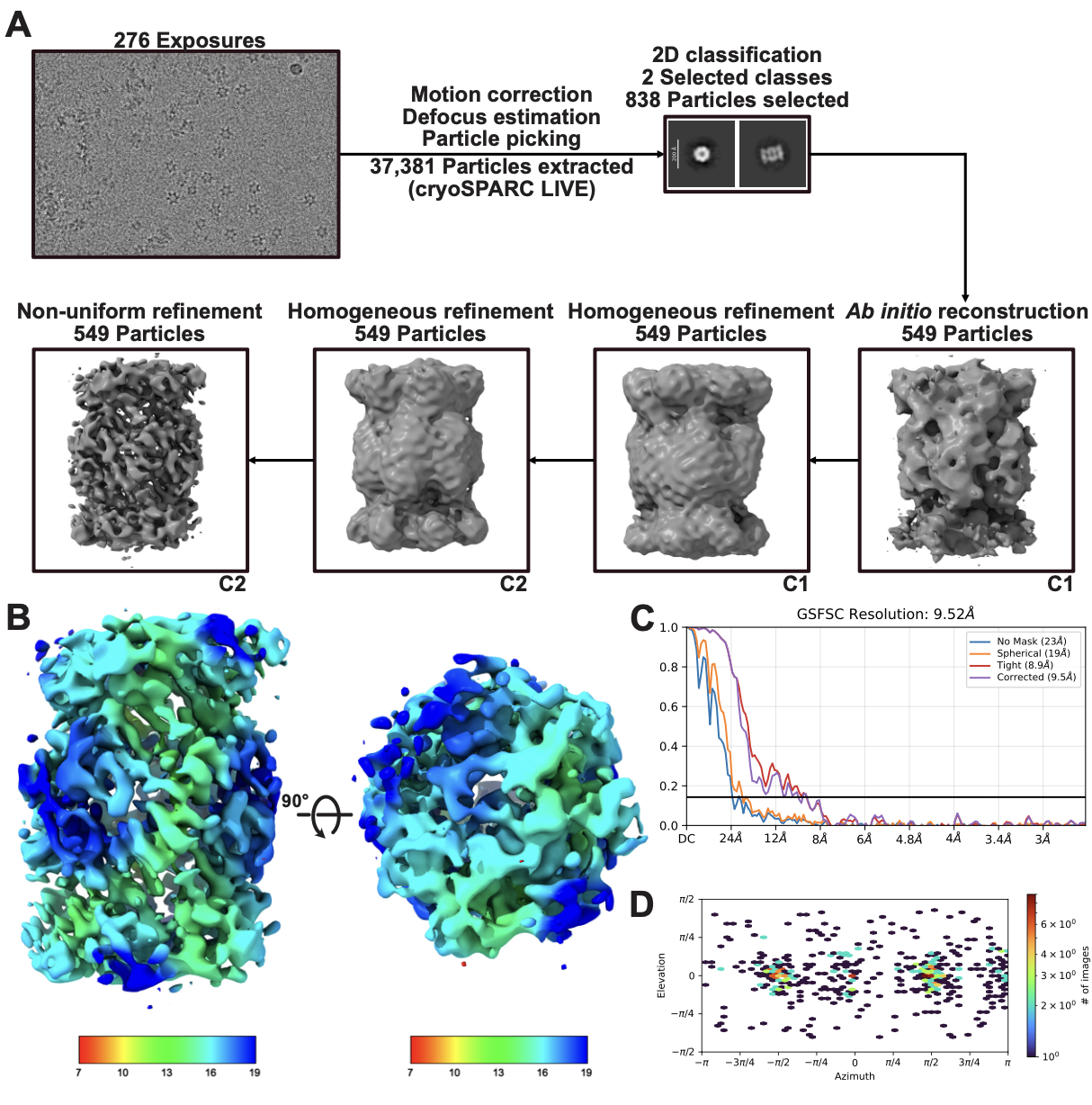
**

**Figure S4. Cryo-EM processing pipeline for the *D. discoideum* 20S proteasome.**


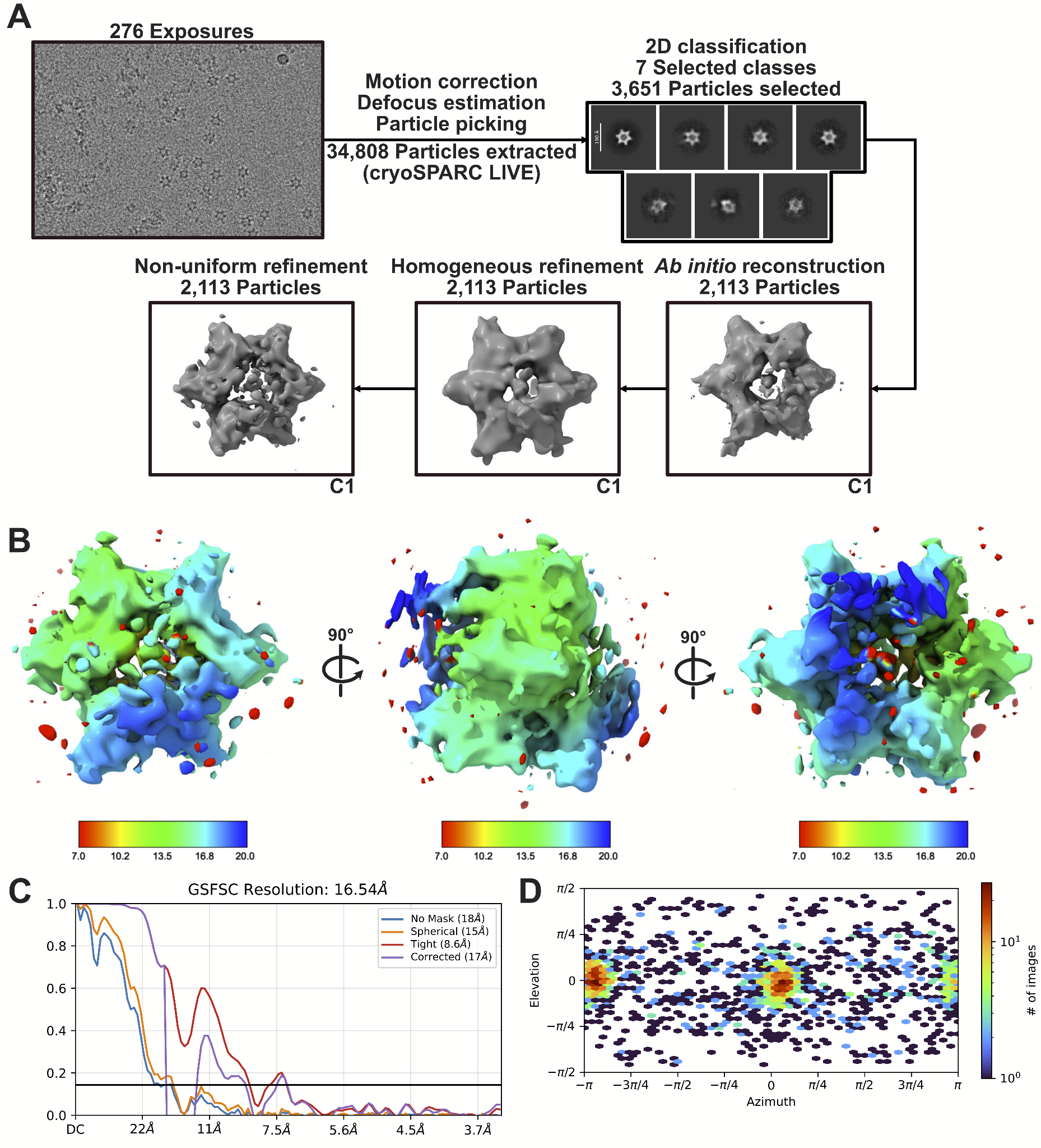


**Figure S5. Cryo-EM processing pipeline for the *D. discoideum* hexameric star complex.**

**
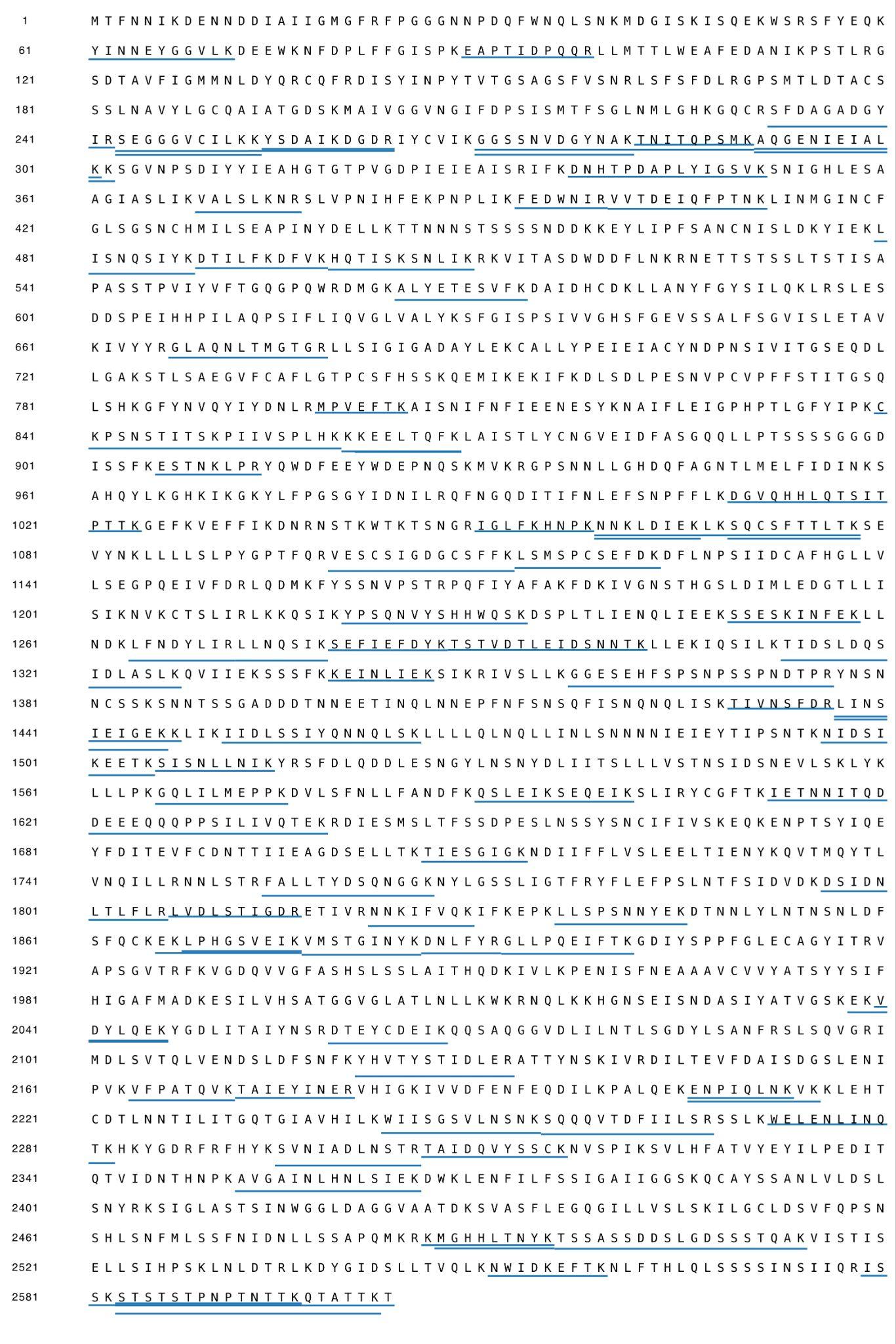
**

**Figure S6. Mass spectrometry-identified peptides for Pks16.**

**
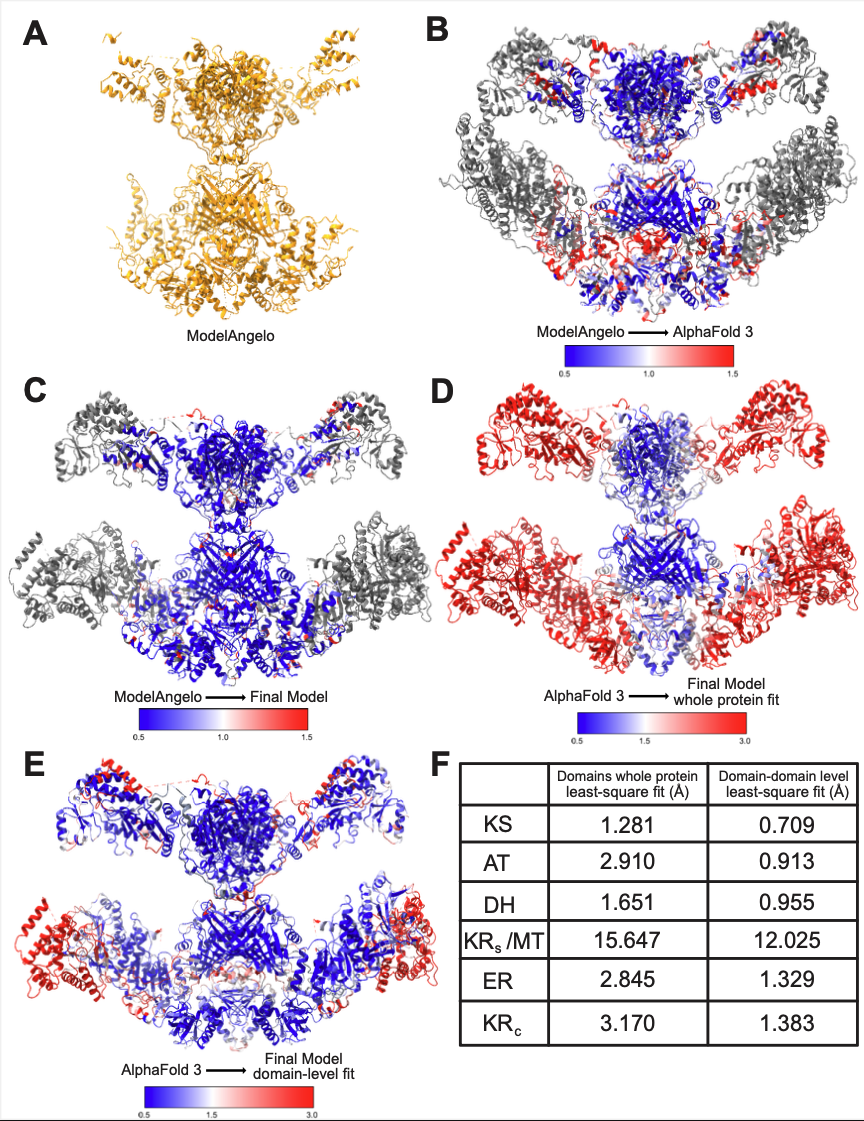
**

**Figure S7. Comparisons between ModelAngelo-built model, AlphaFold model, and final refined model of Pks16.**

**
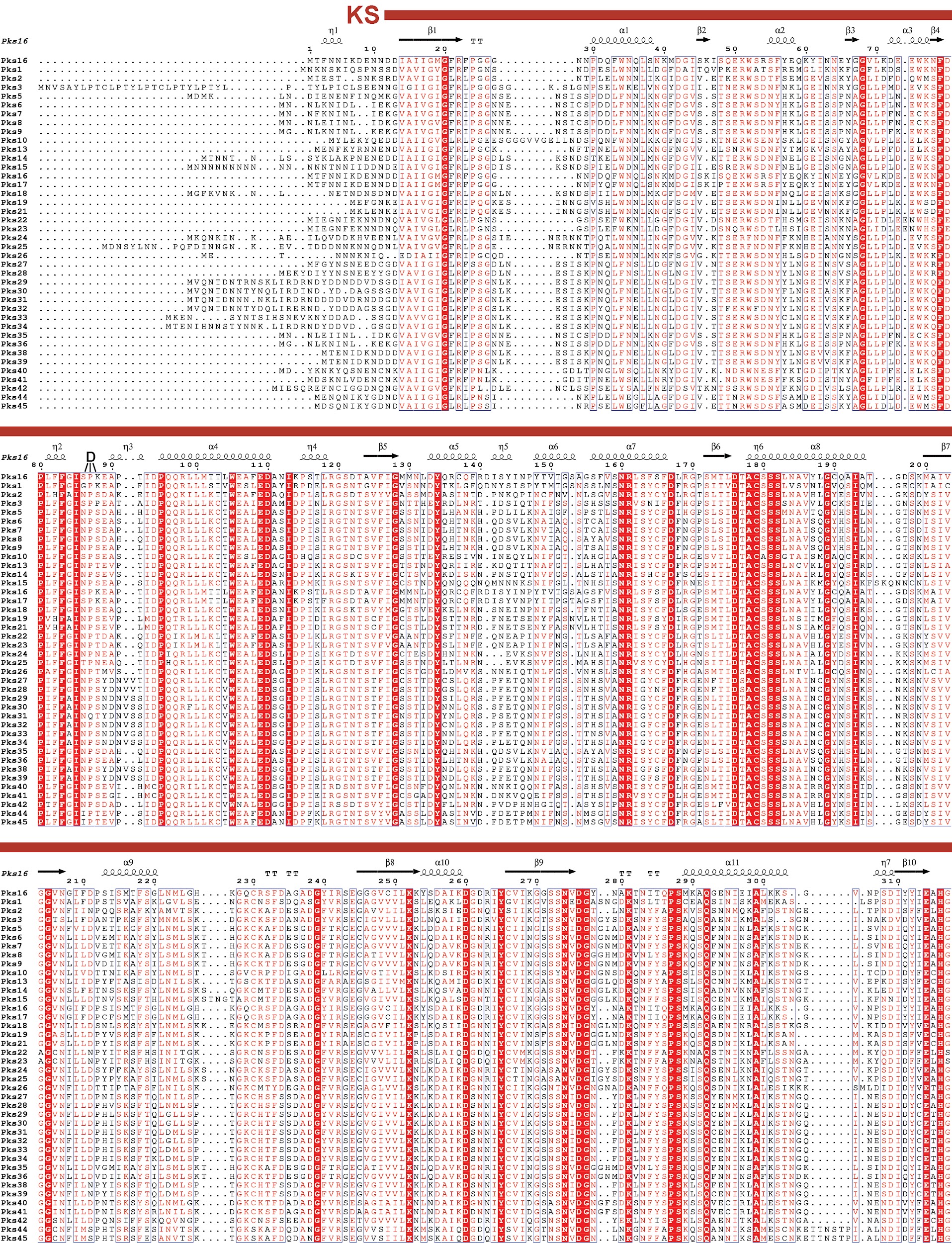
**

**
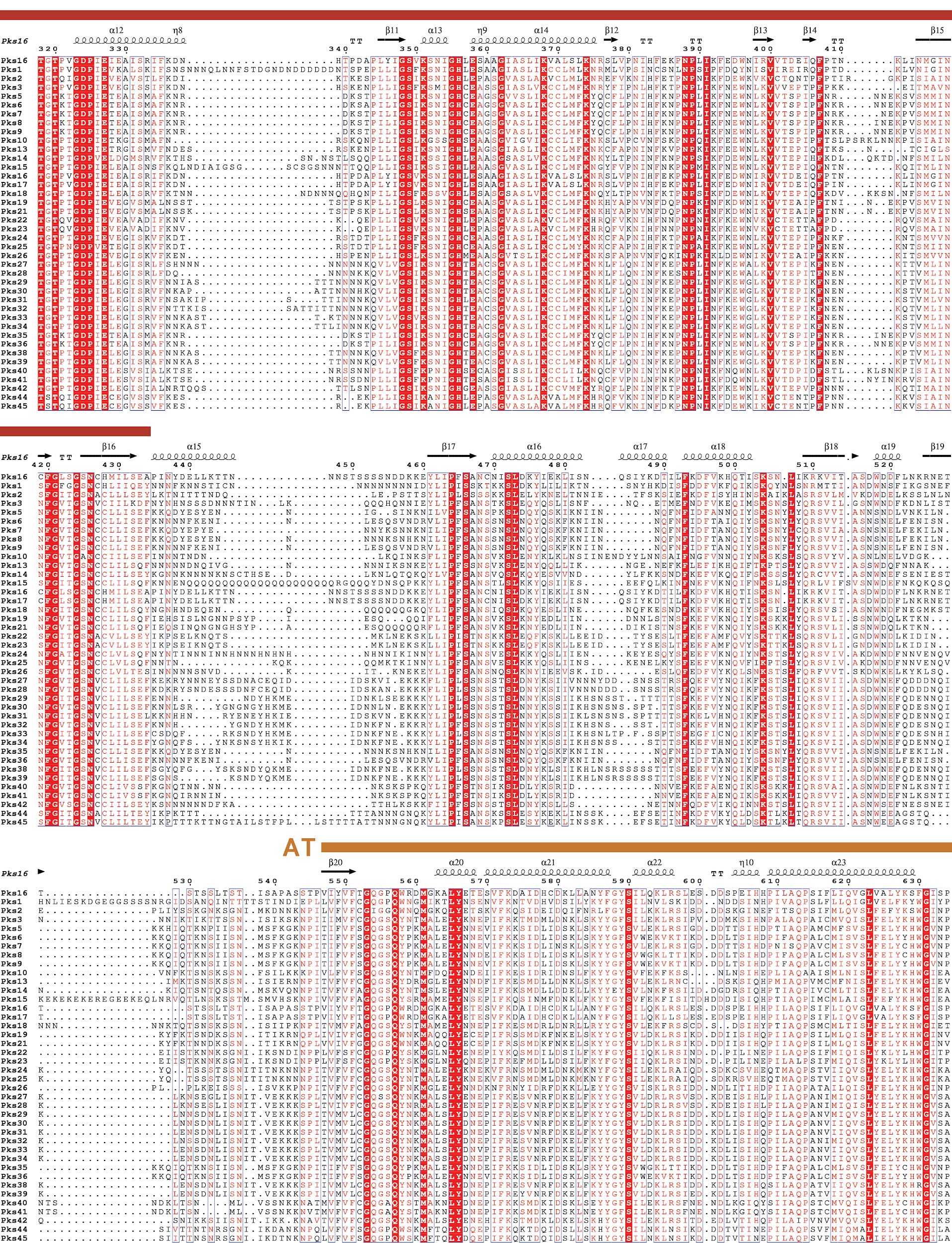
**

**
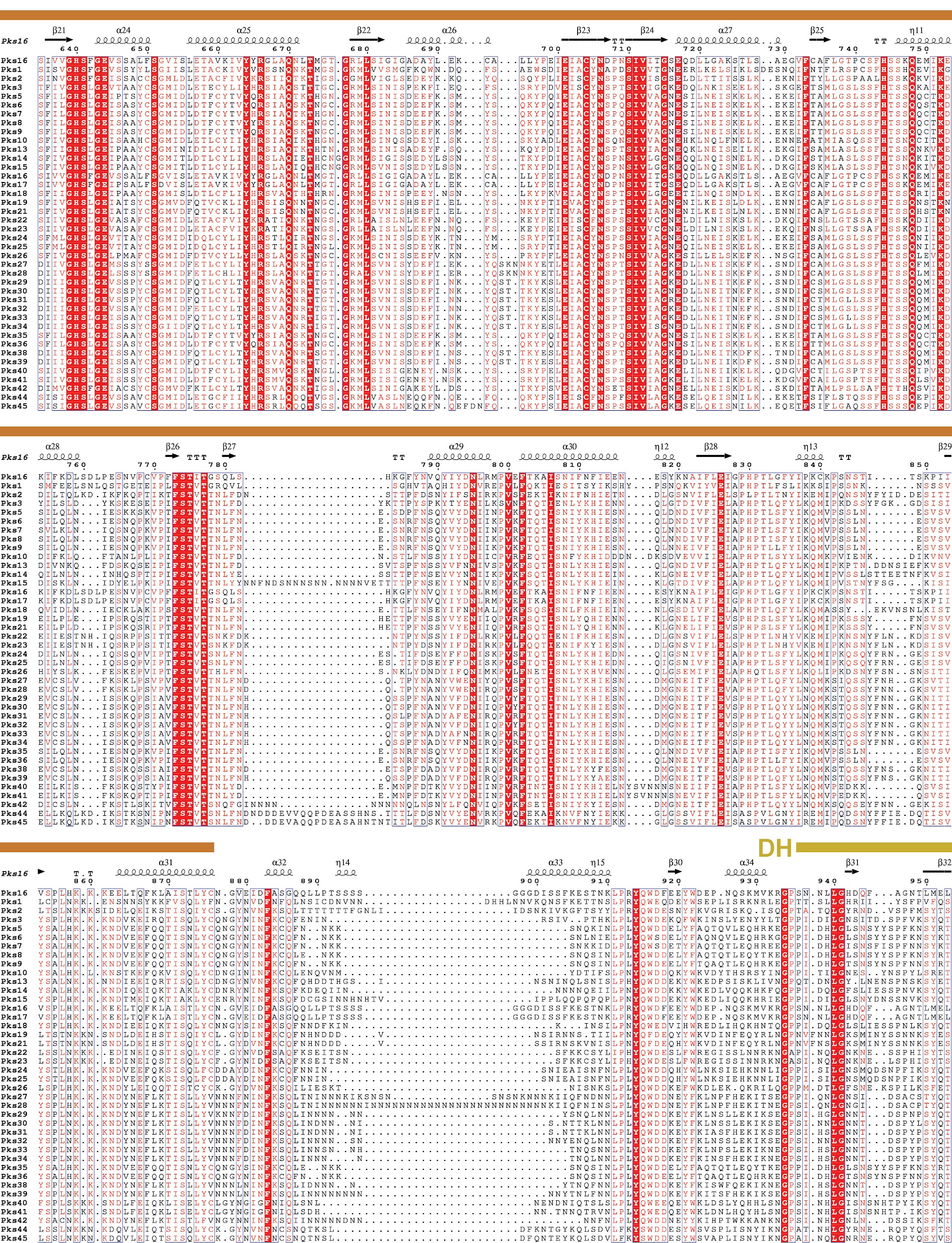
**

**
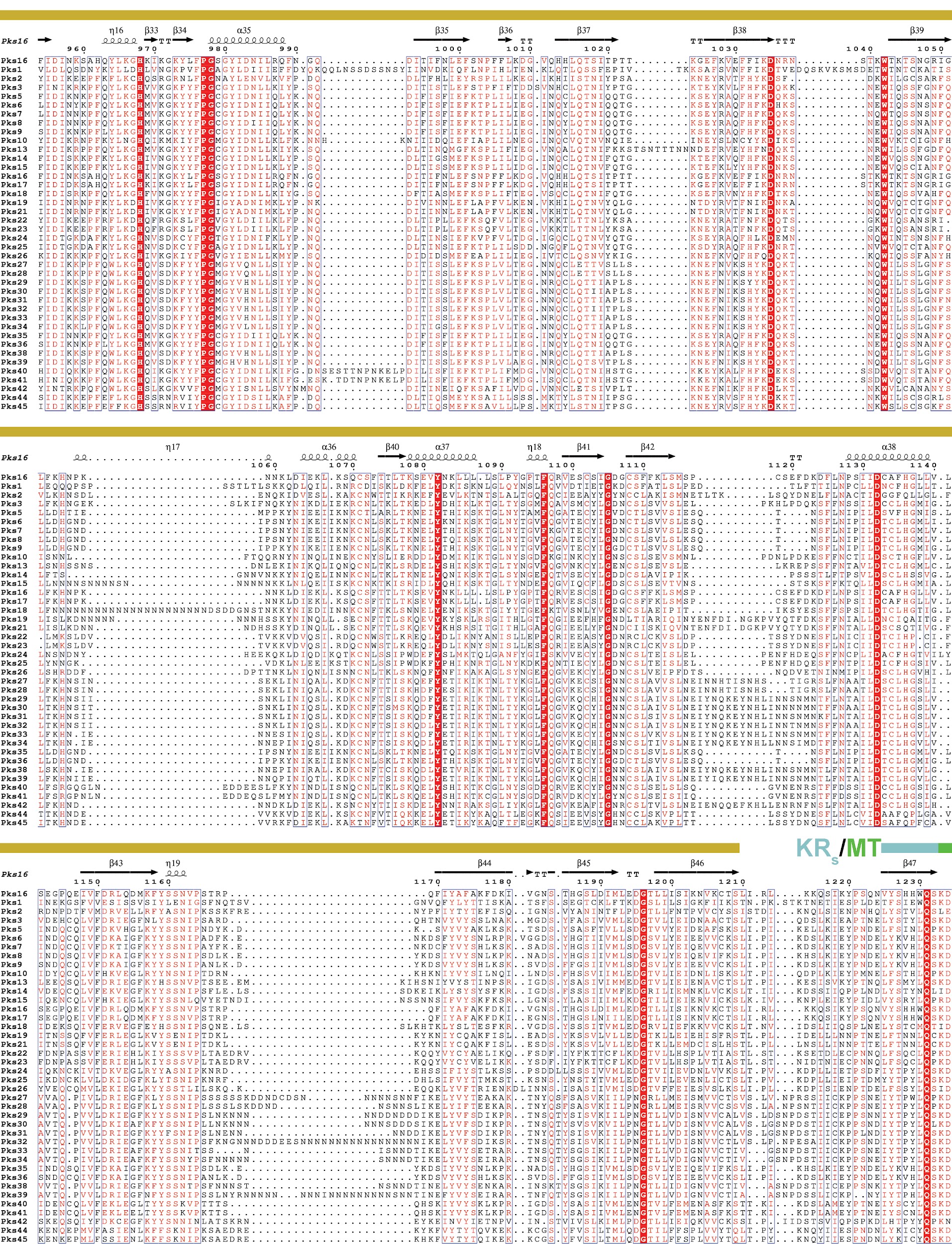
**

**
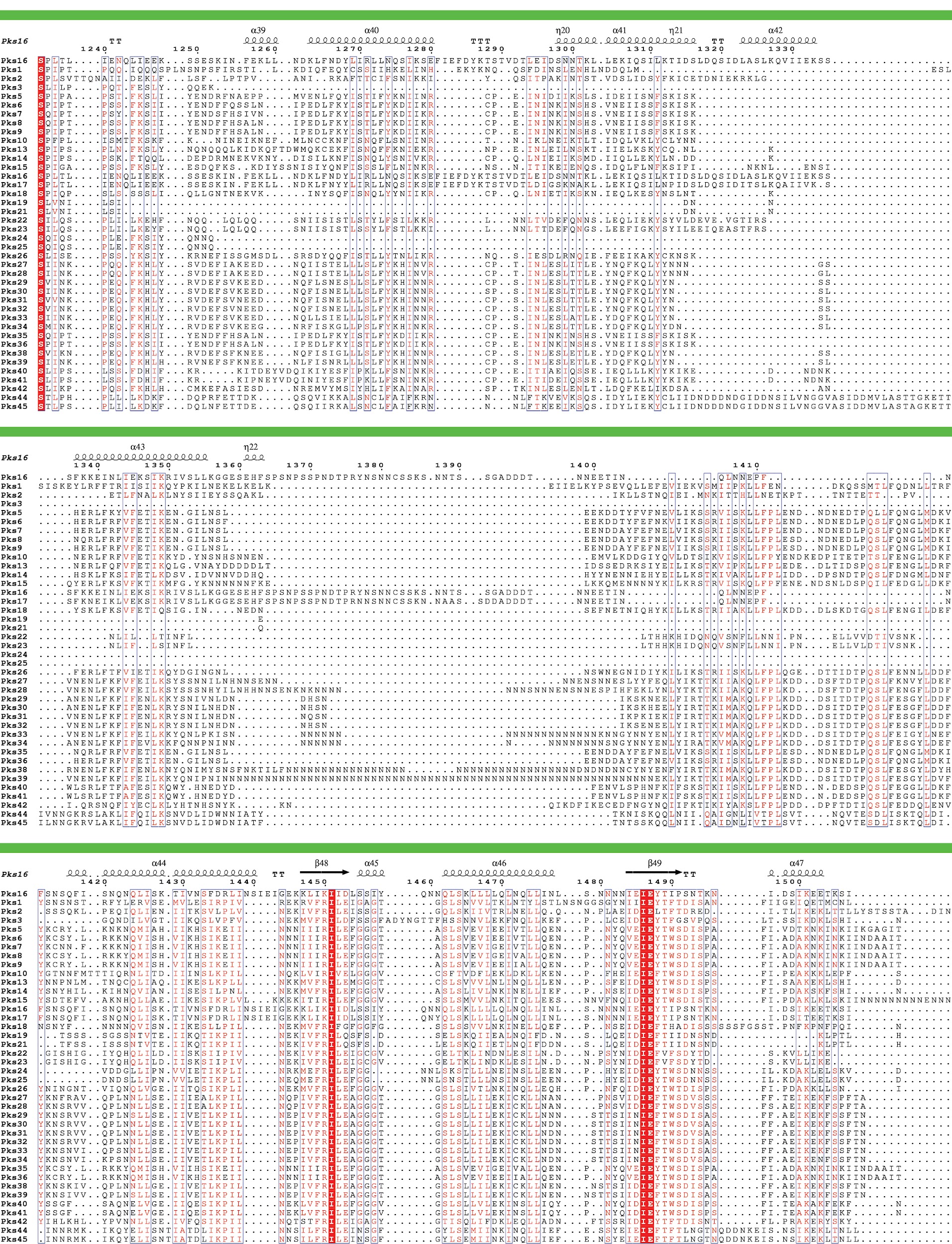
**

**
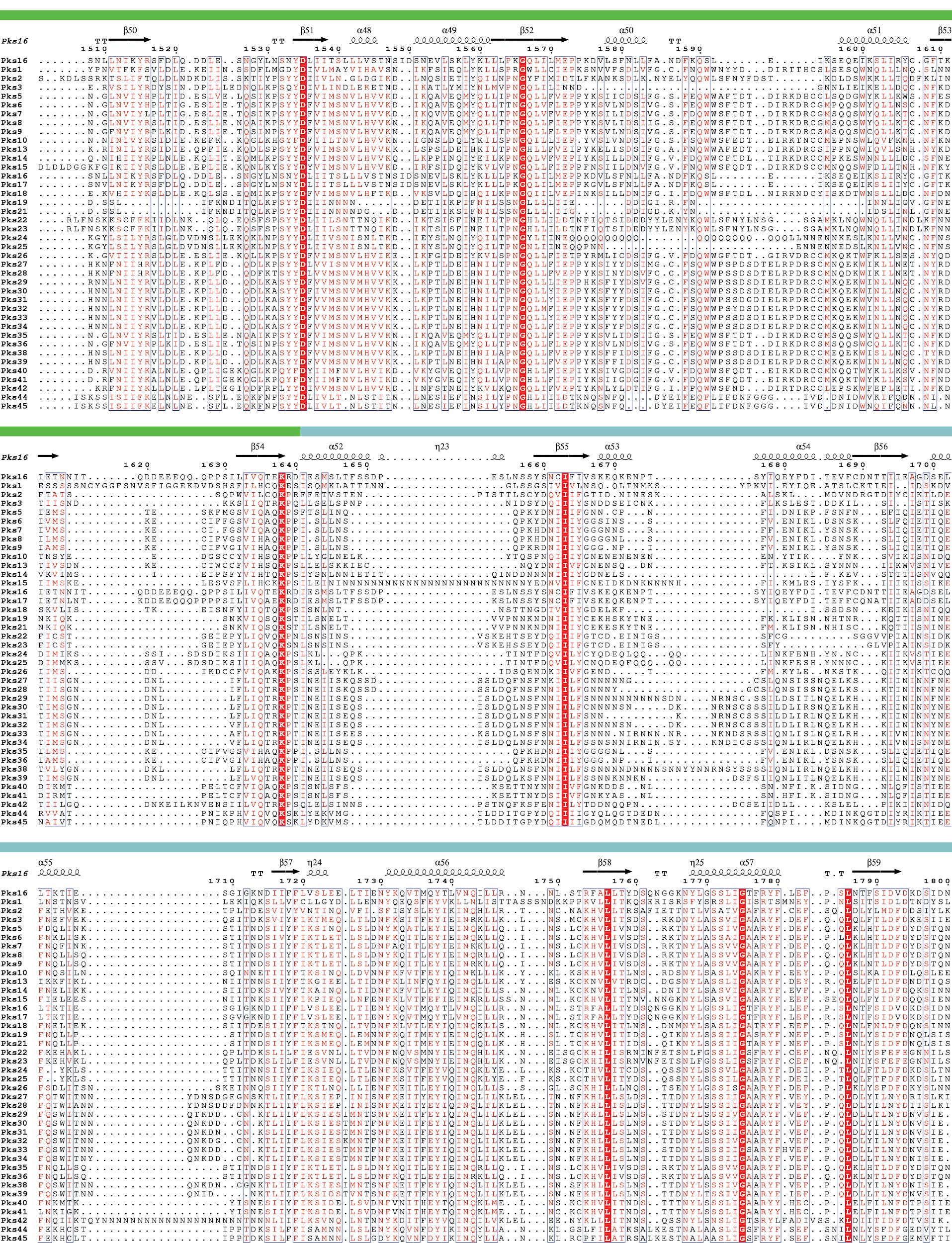
**

**
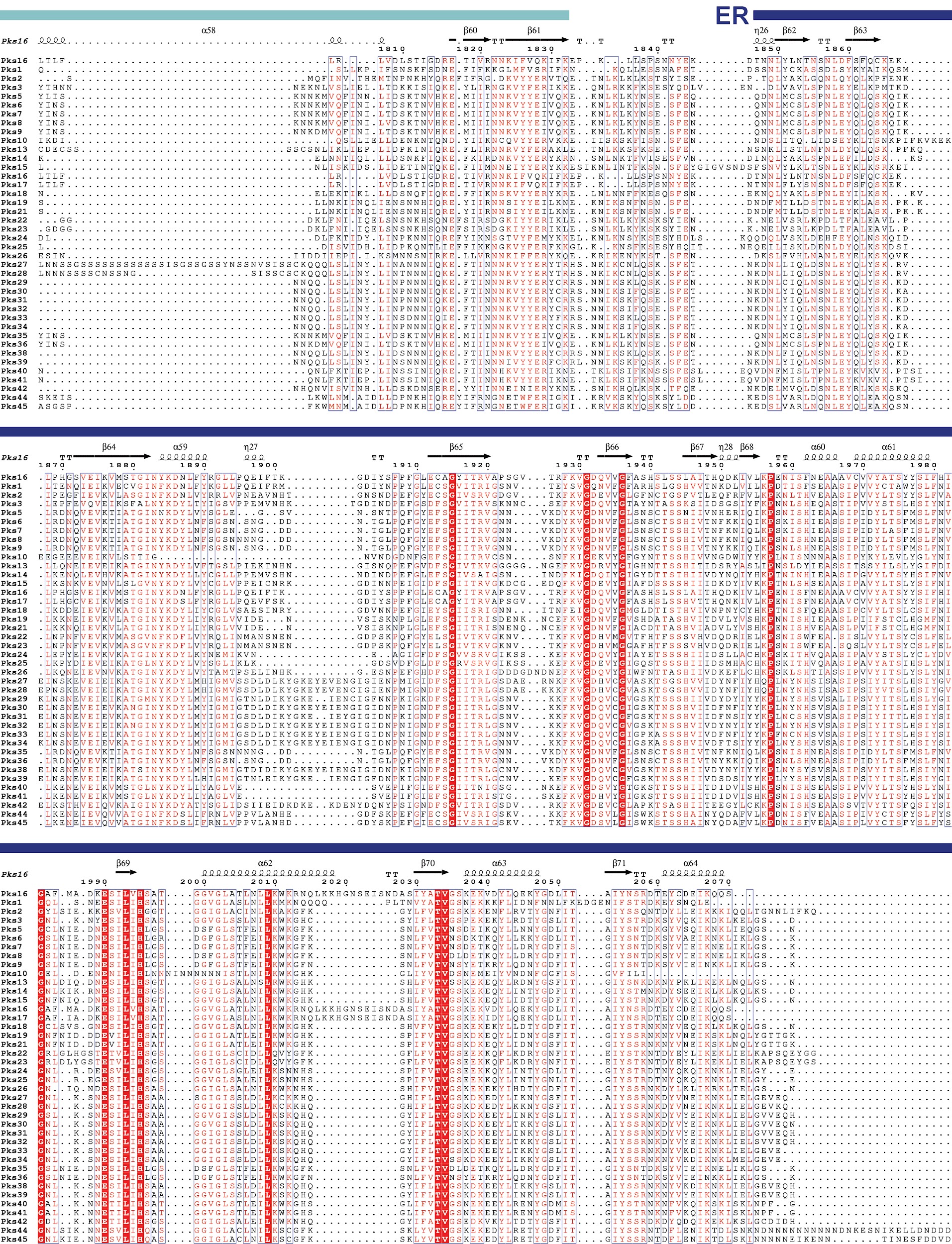
**

**
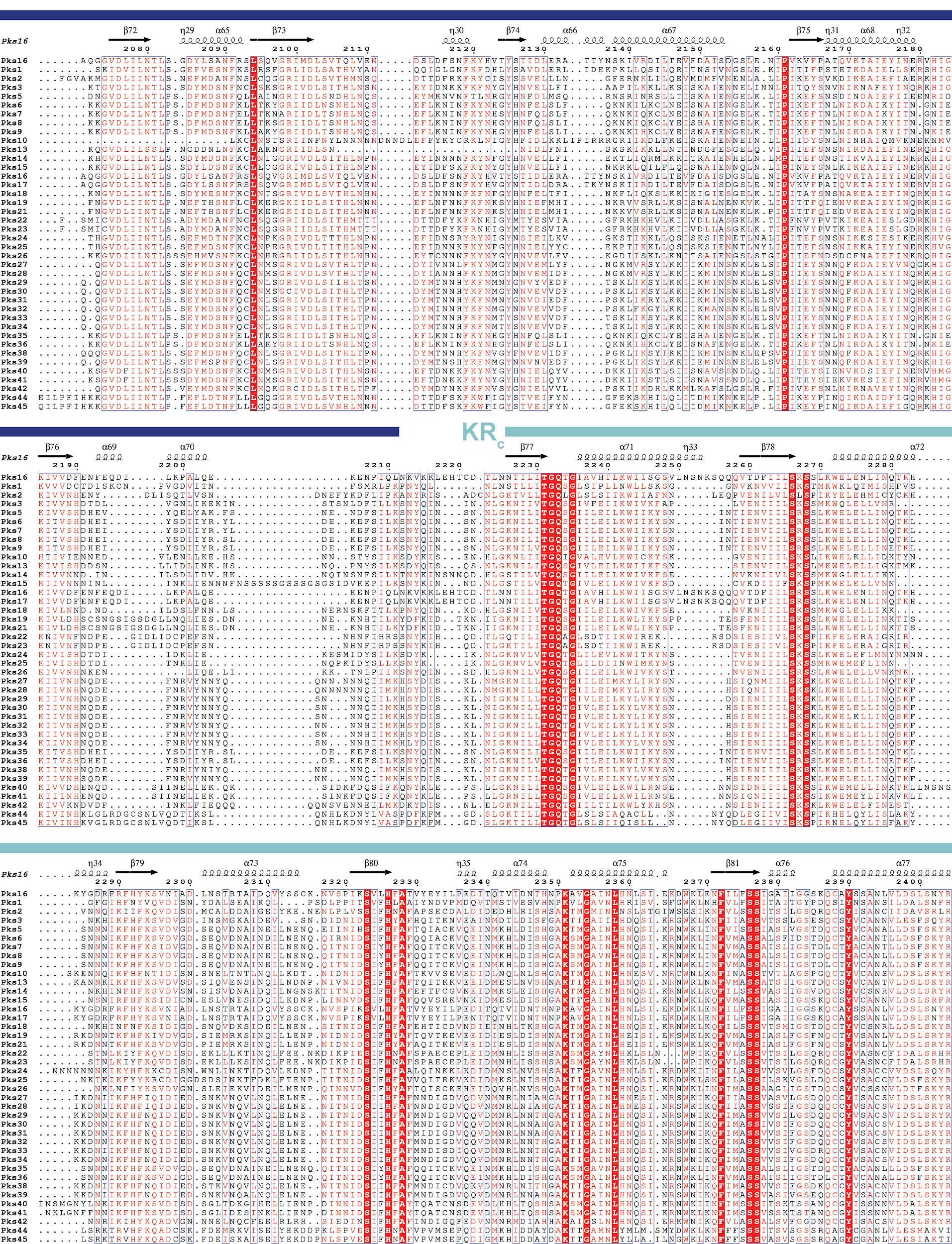
**

**
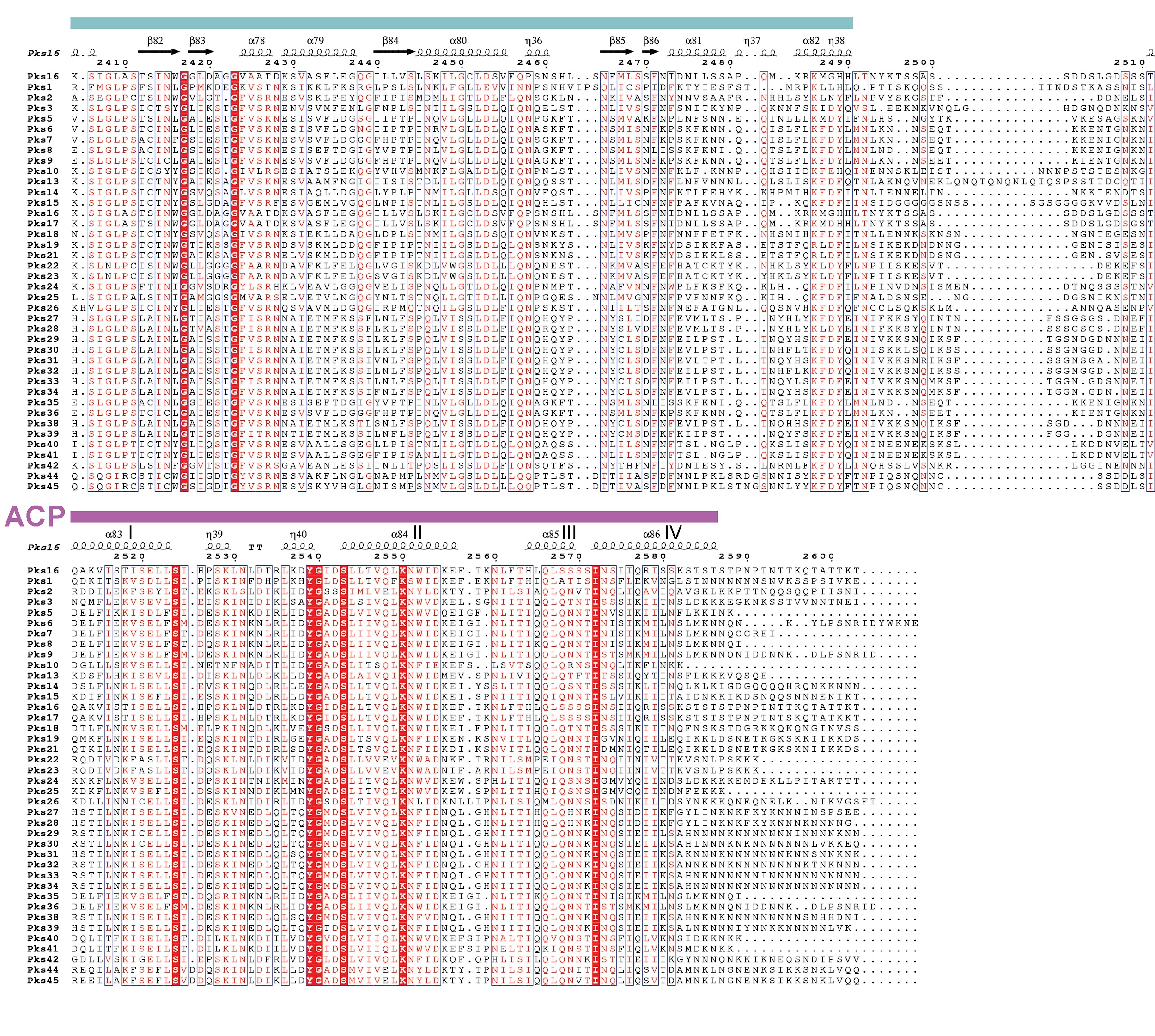
**

**Figure S8. Multiple sequence alignment for the KS-ACP regions of the *Dictyostelium discoideum* PKSs (except Pks37).**

**
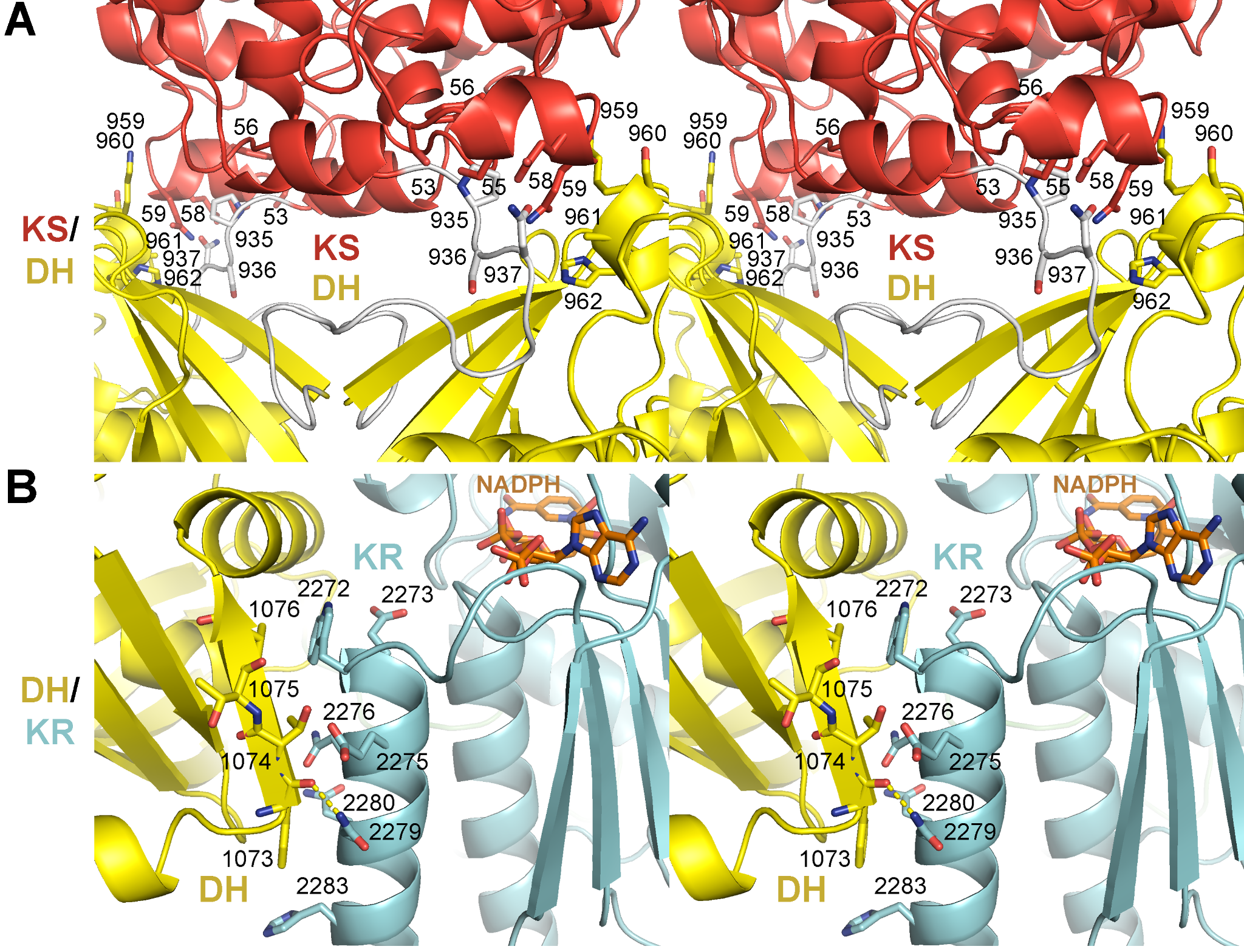
**

**Figure S9. Structural interfaces between Pks16 domains.**

**
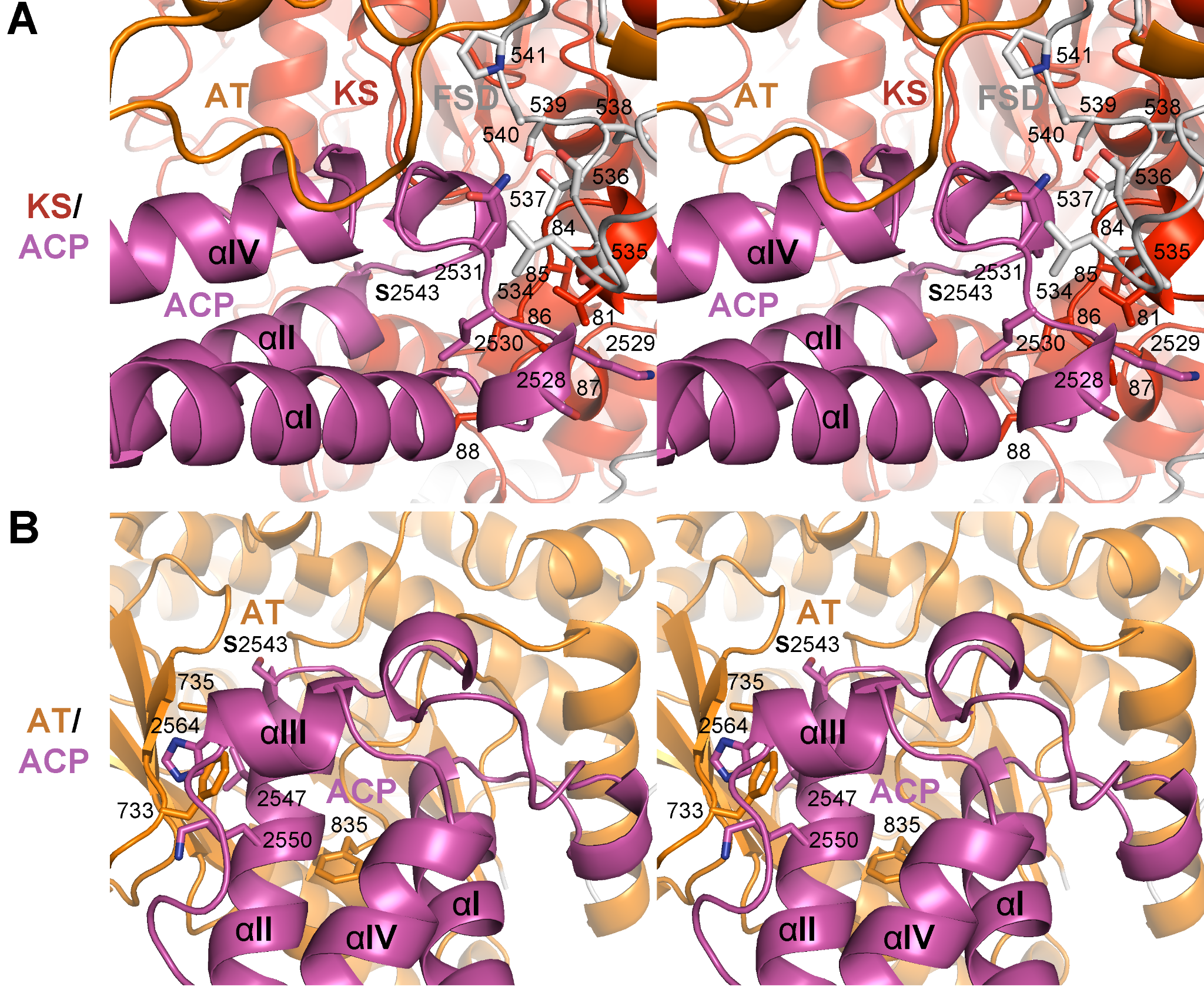
**

**
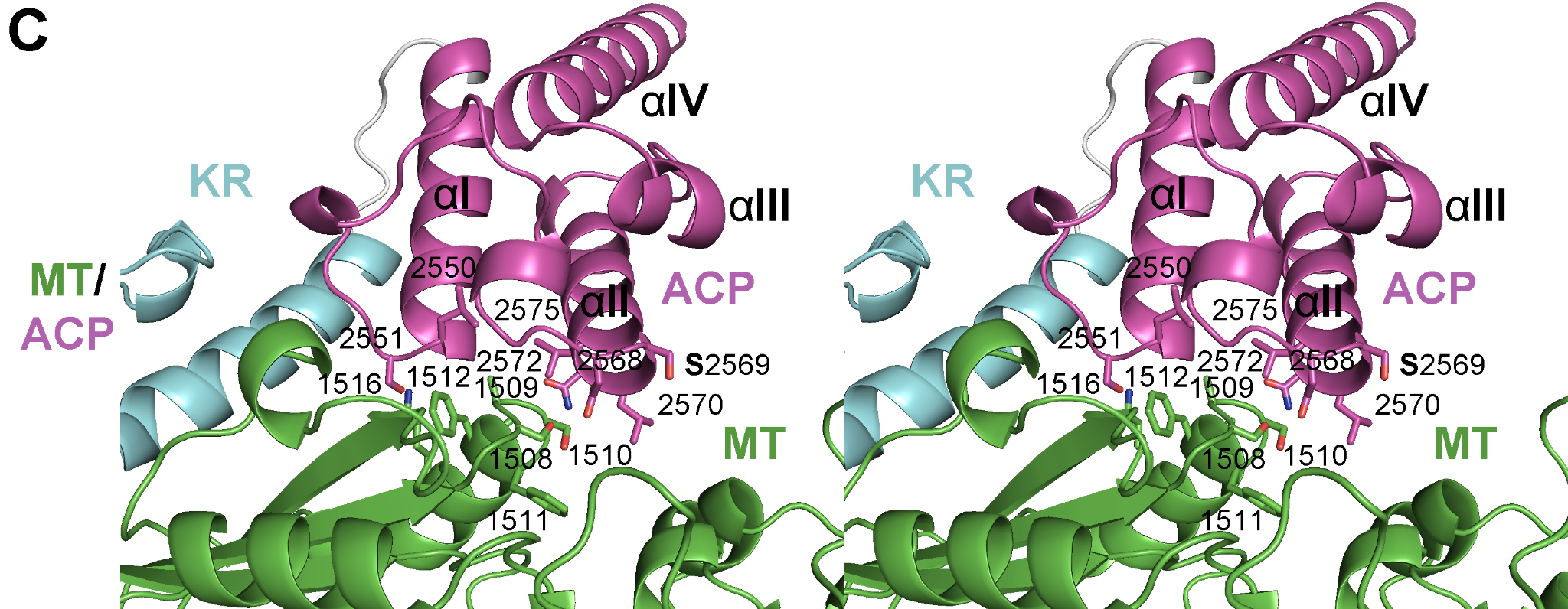
**

**
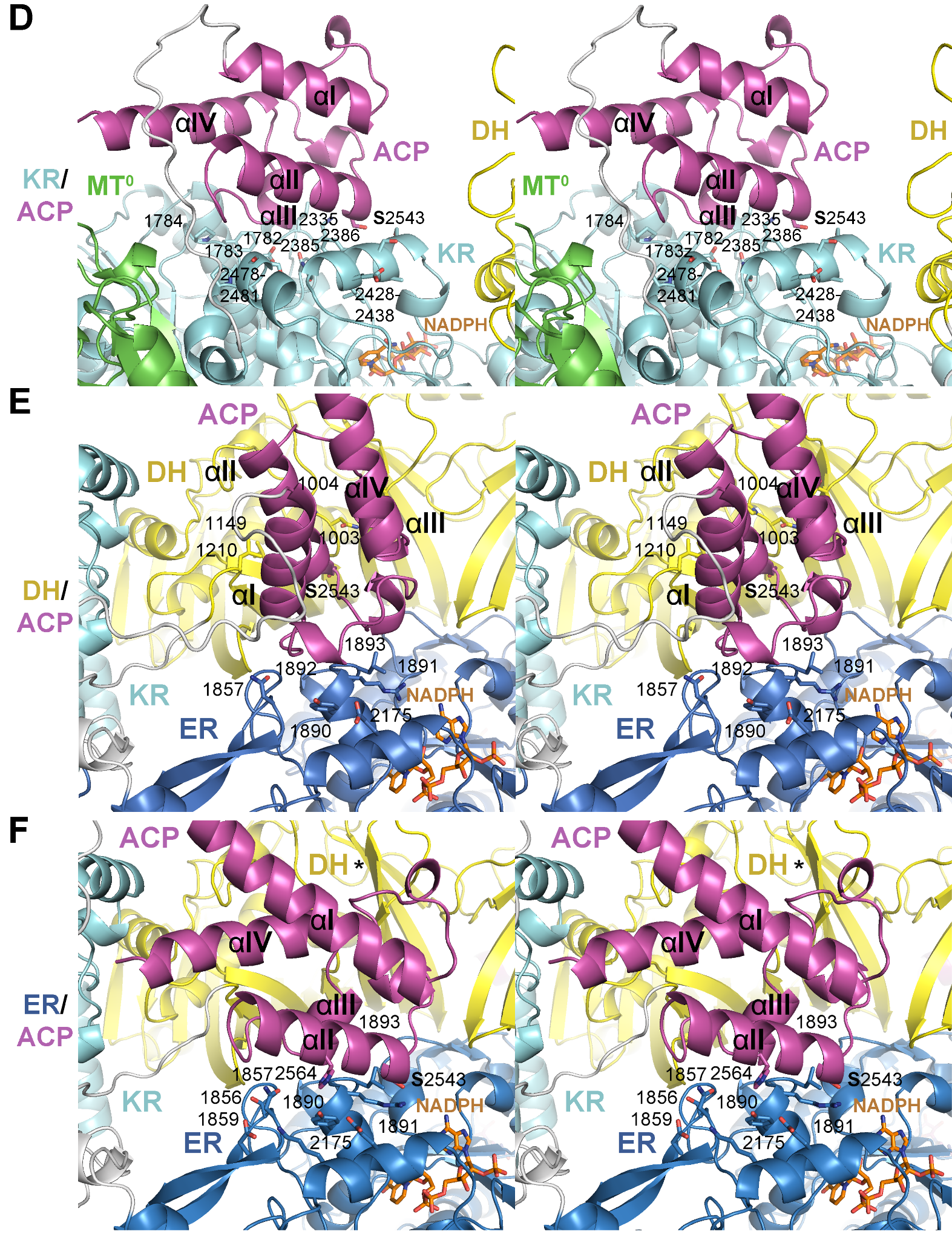
**

**Figure S10. ACP docking sites.**
